# Viola ergonomics for thriving and health promotion: the influence of an instrument’s dimensions, positioning, and biomechanics on muscular effort

**DOI:** 10.3389/fpsyg.2026.1767988

**Published:** 2026-04-10

**Authors:** Oliver Margulies, Martin Faller, Matthias Nübling, Joshua Avery, William Verheul, Wulf Hildebrandt, Horst Hildebrandt

**Affiliations:** 1 Music Physiology/Musicians’ and Preventive Medicine Section, Department of Music, Institute for Music Research (IMR), Zurich University of the Arts (ZHdK), Zürich, Switzerland; 2 Swiss University Centre for Music Physiology, Basel/Zurich, Switzerland; 3 GDB mbH, Data and Consulting, Denzlingen, Germany; 4 Institute for Anatomy and Cell Biology, Philipps-University Marburg, Marburg, Germany; 5 Basel Academy of Music, University of Applied Sciences and Arts Northwestern Switzerland, Basel, Switzerland

**Keywords:** biomechanics, electromyography, muscular effort, musicians’ medicine, prevention, viola ergonomics

## Abstract

**Background:**

The great variability of viola dimensions is known. Contrary to the violin, a lack of scientific knowledge remains on how dimensions, positioning, and biomechanical parameters contribute to the high incidence of medical complaints in violists.

**Aims:**

This project investigated how an instrument’s dimensions affect objective and subjective levels of muscular effort in a player’s left hand and arm, together with the instrument’s position and biomechanical parameters.

**Methods:**

In a laboratory setting, a quantitative, intra-individual comparison between two violas, V1 and V4, predefined in dimensions and positions, and the participants’ own instrument was carried out. Muscle activation (EMG) and perceived effort (BORG) of 31 violists (15 m/16w, mean age 24.8 years, SD = 3.6) were recorded while playing a 16-s tune. Measurements with instruments V1 and V4 were conducted in standardized positions (A1, A2, B1, B2) without and with the players’ own instruments with ergonomic aids. EMG/BORG data were associated with individual biomechanical parameters (BHM).

**Results:**

Positional changes of the instruments’ longitudinal and lateral axes significantly affect target parameter values. Between the extreme positions A1 vs. B2, the mean delta for EMG decreased 29% relative to A1 (*p* < 0.001), and for perceived effort (BORG) 28% (*p* < 0.001). Comparing the instruments V1 and V4 shows significant differences for BORG (*p* < 0.05) but not for EMG at this data aggregation level. A key finding for biomechanics is the negative correlation between all arm length parameters with EMG and BORG (both *p* < 0.001).

**Conclusion:**

Results for the viola reconfirm that instrument positioning affects muscle activation and subjectively perceived effort, highly significantly. This expands possibilities to deduce individualized ergonomic solutions, benefiting playing posture, practicing, and performance. The prevention and solution of Playing-Related Musculoskeletal Disorders (PRMDs) could be facilitated, permitting this group of musicians to thrive. Future sub-analysis aims to identify instrument build effects at the single-muscle level and for the hand positions under test. Further research in real-life settings will be needed to understand how longer durations of playing affect the same target parameters.

## 1 Introduction/background

Over the last decades and internationally, musical institutions both at the training and professional levels are demonstrating a rising awareness of health problems linked to music-making. First publications documenting the high prevalence of task-specific – in some cases multiple – health problems in orchestra musicians can be traced back to the 1980s (Fishbein et al., 1988). These findings have led to a growing number of initiatives benefitting musicians with the targeted prevention, diagnosis, and treatment of task-specific complaints (Alessandri et al., 2024; Hildebrandt, 2009, 2015; Spahn et al., 2001). With figures largely unchanged since research in this field began and against the background of time and effort necessary to achieve the required degree of proficiency for professionalism (Ericsson et al., 1993), epidemiological examinations identified complaints in the musculoskeletal system, the ear, and the field of psychosomatics in approximately 75% of all professionals and more than 50% of musicians in training (Ackermann and Adams, 2003; Bächinger et al., 2022; Cruder et al., 2020; Hildebrandt, 2002; Hildebrandt et al., 2012; Kenny and Ackerman, 2013; Rosset et al., 2022; Rotter et al., 2020; Spahn et al., 2002; Williamon and Thompson, 2006; Wu, 2007).

Due to one-sided posture and movement patterns typical for players of high stringed instruments, strains on the upper extremities are among the most common task-specific health problems (Ackermann and Adams, 2003; Berque and Gray, 2002; Blum, 1995a; Blum, 1995b; Kok et al., 2015; Moore et al., 2008; Nyman et al., 2007; Rousseau et al., 2023; Steinmetz et al., 2008; Vinci et al., 2015; Steinmetz and Goar-Oberwesel, 2016), particularly on the left side (Möller et al., 2018; Rensing et al., 2018; Robitaille et al., 2018; Schemmann et al., 2018; Shan et al., 2004). Despite a steadily growing number of publications with a focus on this specific epidemiological detail, the focus on individual physical predispositions in relation to the instrument played remains a niche in research in general (Obata and Kinoshita, 2012; Rabuffetti et al., 2007; Seidel et al., 2009; Visentin et al., 2015; Spahn et al., 2014; Steinmetz et al., 2006; Storm, 2006; Zaza and Farewell, 2001), as does electromyographic measurements of muscle activity specifically (Berque and Gray, 2002; Cattarello et al., 2017; Fjellman-Wiklund et al., 2004a, 2004b; Mann et al., 2021, 2023; McCrary et al., 2016; Möller et al., 2018; Rensing et al., 2018; Schemmann et al., 2018; Shan et al., 2004).

A number of publications consider unfavorable body and instrument proportions as risk factors for overuse syndromes of the musculoskeletal system (Hildebrandt, 2000; Klashorst, 2002; Lahme, 2000; Seidel et al., 2009; Smithson et al., 2017; Ramella et al., 2014; Rensing et al., 2018; Schemmann et al., 2018; Shan et al., 2004; Visentin et al., 2015). With the exception of research results from our team (Hildebrandt, 2017, 2018; Hildebrandt et al., 2016a, 2016b, 2017, 2021; Hildebrandt and Margulies, 2018; Margulies et al., 2023), the mention of possible individual limitations or predispositions in instrument-specific biomechanics of the upper extremities could only be tracked down in a limited number of publications (Chi et al., 2020; Hüwe and Träder, 2015; Kelleher et al., 2013; Margulies and Hildebrandt, 2014; Visentin et al., 2015). Regarding the influence of a viola’s dimensions on muscle activity and subjectively felt effort and its influence on the development of task-specific health problems, hardly any literature could be identified prior to this study (Blum, 1995a; Blum, 1995b; LeVine and Irvine, 1984).

Viola literature repertoires document important compositions for this instrument spanning several centuries (Ewald, 2013; Lainé, 2010). Technically challenging compositions were written for it, some tailored to their composers’ or a specific performer’s technical and artistic aptitudes (Ewald, 2013; Weaver, 2006). Research suggests that this may pose challenges for other violists, whose hands’ characteristics differ from the first groups mentioned (Margulies and Hildebrandt, 2014; Wagner, 2005; Wohlwender, 2009). The importance of considering a viola’s ergonomic aspects is underpinned by the instrument’s great variations in its dimensions compared with the violin. Based on the classical proportions of the instrument (Möckel, 1990; Sacconi, 1981), expert luthiers consider a viola to be within the norm even if the ranges show much greater differences than in the case of the violin (cf. Supplementary Table S1).

Shifting our focus to the viola arose from experience gathered within the framework of musicians’ health counseling sessions: Despite a growing body of knowledge available, task-specific health problems in violists often appear to arise due to a musician-instrument-misfit. Although research on the acoustical characteristics of the violin and viola suggests otherwise, an erroneous belief persists among violists that instruments with a larger body length produce a greater sound. For violists, the choice of an ergonomically and acoustically disadvantageous instrument may have far-reaching consequences regarding the emergence of task-specific health problems. Although the systematic, individual consideration of a player’s biomechanics relative to the instrument is a more recent development (Blum, 1995a; Blum, 1995b; Hildebrandt et al., 2016a, 2016b, 2016c, 2017, 2021; Margulies and Hildebrandt, 2014; Margulies et al., 2023; Wagner, 2005, 2012), no data specifically for the viola were available prior to this study. Also, only more recent advances in research carried out on the acoustics of string instruments are paving the way toward individually tailor-made instruments uniting optimal acoustic and ergonomic characteristics of stringed instruments.

## 2 Aims, research question, and current status of research

### 2.1 General aims

In keeping with our previous research (Hildebrandt, 2017; Hildebrandt et al., 2016a, 2016b, 2017, 2021; Margulies et al., 2023), this project aims to expand the so far available scientific foundation of individualizing ergonomic approaches to instrument positioning and playing with a specific focus on the viola. We thereby aim to contribute to the growing body of knowledge of how to prevent task-specific health problems, to offer solutions for violists to thrive throughout their career path, and to shorten recovery times in case of task-specific injury. Our research aims to yield supportive measures benefiting not only the performers themselves, but also specialists interacting with musicians, such as teachers, instrument makers, specialists in the field of music physiology, and therapists.

### 2.2 Research question

Our research aimed to investigate how an instrument’s dimensions affect levels of muscular activation and subjectively perceived effort in a player’s left hand and arm in conjunction with the instrument’s playing position and individual biomechanical parameters.

### 2.3 Status of the research

Research results presented in this paper build on our first results for the violin (Hildebrandt et al., 2021, Margulies et al., 2023). Focusing on violin positioning effects on physiological parameters and the degree of compensation movements in a player’s left upper extremity, our findings were that, when playing a predefined, 16-s tune in two different hand positions, both the levels of muscle activation (EMG) and perceived effort (BORG-scale) showed changes depending on how the instrument was positioned relative to the player: Mean values of overall muscle activation in the *M. pectoralis* major, *M*. *biceps brachii*, *M*. *extensor carpi ulnaris*, and *M*. *extensor digitorum communis* muscles (with particularly pronounced effects for the *M. pectoralis* muscle) and of BORG values increased significantly and independently in the violinist’s left arm, the nearer the instrument’s longitudinal axis was relative to the player’s central sagittal plane, and the nearer the instrument’s lateral axis relative to the player’s horizontal plane (Hildebrandt et al., 2021). Equally, our research demonstrated that pre-existing biomechanical factors in conjunction with the instrument’s positioning relative to the player are likely to determine the degree of compensation movement of a violinist’s left upper extremity when playing the instrument. This was shown by collecting data by means of the Biomechanical Hand Measurement according to Wagner (2005, 2012), 3D motion capture analysis, and 2D HD video monitoring for the players’ elbow and upper arm adduction, as well as for shoulder elevation and protraction, with protraction being more pronounced than elevation (Margulies et al., 2023).

An important basis for this study is the research contributed by Wagner (Schuppert and Wagner, 1996; Wagner, 1977, 2005, 2012; Wilson et al., 1991, 1993). First objective evidence of the wide range of individual supination capacity was provided by surveys and a comparative study between non-musicians, orchestra musicians, and participants in a large international violin competition (Wagner, 2005). It was shown that considerable differences exist in the ability of *passive* supination, an indicator of the ease of movement due to joint and tissue resistance in the left hand and forearm. A player ranging at 90 degrees passive supination (lateral wrist axis nearly horizontal) as a result of a light torque (16 Ncm) differs considerably from one ranging at 30 degrees with the same torque. Those with a lower degree of passive supination (30°) must actively supinate (Mm. biceps and supinator) earlier and more to assume the playing position. Passive supination ability generated by external torque is particularly relevant, as differences are less obvious in *active* supination. Routine clinical examinations often record normal values for *active* supination, but significant deficits in *passive* supination with little torque remain unobserved. Such individual “movement brakes” in joint structures and tissue properties may provoke players to “force” themselves into a required position. This is done by engaging muscles involved in a playing motion more, particularly in the bicep muscle and, less clearly definable and subjectively perceptible, in the supinator muscle. Actively overcoming these “movement brakes” can lead to coordinative and later muscular fatigue in the muscles involved, with disruptive influences on wrist and long finger musculature originating near the elbow. Fatigue in the forced supination position may originate from position-related, direct increase in tone in wrist and finger muscles and from friction contacts in the lower arm compartment and contact with the tensed bicep insertion tendons (e.g., lacertus fibrosus). These relationships may explain differences between competing players of high-stringed instruments: Apart from the basic positions of the instrument and hours of exercise for decades, ease, speed, effortlessness, and stamina are key parameters in virtuoso playing. Such externally invisible “movement brakes” must be viewed as co-factors of reduced playing efficiency and overuse complaints occurring at an early stage in players of high-stringed instruments of all ages and have previously been hard to explain (Hildebrandt, 2006; Wagner, 2005, 2012). Regarding the viola, this study focused on the interdependency between the aforementioned passive supination and further biomechanical target parameters and the viola’s build in terms of its string length and body dimensions, therefore adding to the complexity of gaining an accurate understanding of how the player’s individual hand characteristics and the instrument’s target parameters interact systemically.

### 2.4 Hypotheses

For our research, the following hypotheses were formulated:

#### 2.4.1 Hypotheses pertaining to the instrument’s dimensions

Hypothesis 1 (main hypothesis): The larger the instrument’s dimensions and its string length, the higher the muscle activation and perceived effort in the violist’s left arm will be when playing.

Sub-Hypothesis 1: The more the instrument’s longitudinal axis points toward the front (relative to the central sagittal plane, LoAx-CSP) and the more horizontal the instrument’s lateral axis (relative to the horizontal plane, LatAx-HP), the more muscle activation and perceived effort will be present in the violist’s left arm when playing.

#### 2.4.2 Hypotheses pertaining to biomechanical parameters

Hypothesis 2: The lower the active and passive supination ability, the more muscle activation and perceived effort will be present in the violist’s left hand and arm when playing.

Sub-Hypothesis 2: (i) The larger the difference in length between the little finger and the middle finger, (ii) the lower values for active and passive spreading between fingers, (iii) the shorter the overall length of the arm and its parts, the more muscle activation and perceived effort will be present in the violist’s left arm when playing.

## 3 Materials and methods

### 3.1 Study design

Our laboratory repeated-measures experimental study included 31 healthy violists (15 m, 16f). Their mean age was 24.8 years (SD = 3.6). The minimum age was 18, and the maximum was 33 years. Study participants were recruited at Swiss music universities, as well as the Stella Vorarlberg Private University College, Austria, and the State University of Music and Performing Arts Stuttgart, Germany (HMDK). The study was carried out with approval of the Canton of Zurich’s Ethics Committee Clarification of Responsibility (BASEC reference number Req-2019-01143). All study participants gave their oral and written informed consent for participation prior to measurement.

### 3.2 Measurement steps

**Figure 1:**
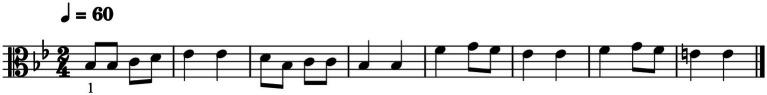
Sixteen seconds tune used for tests.

**Figure 2:**
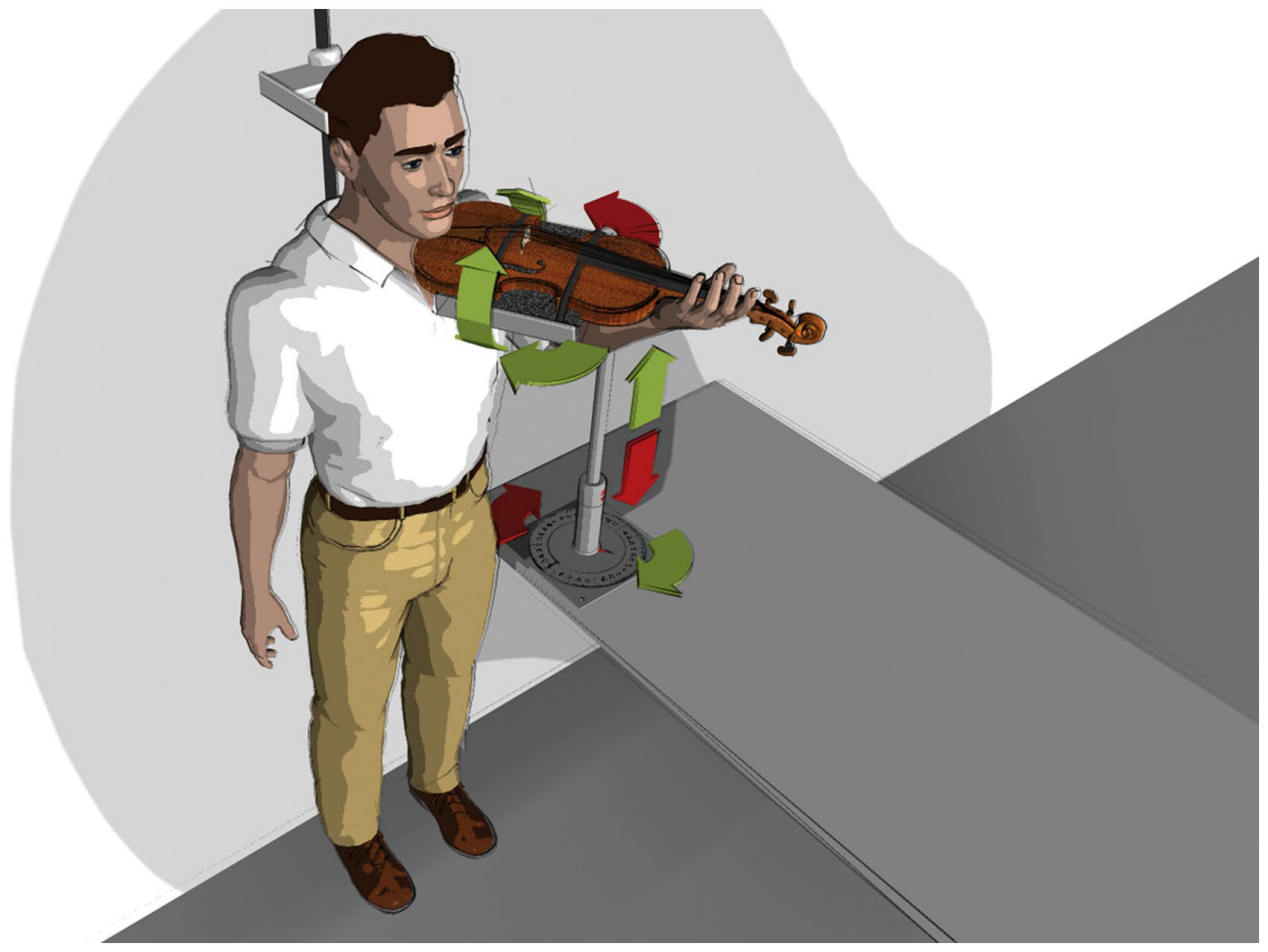
Instrument position standardization (schematic depiction).

- Study participants were asked to play a predefined, 16-s tune (cf. Figure 1) during ongoing comparative muscle activation measurements (EMG) in a laboratory setting. The tune was played by using two violas specifically standardized for the experiment in their dimensions and vibrating string lengths (Table 1 for instrument details). The tune was performed on both laboratory violas in four standardized viola positions in randomized order (see Table 2 for angle descriptions) without the use of ergonomic equipment (see technical prerequisites below).
- The tune was measured identically, but with study participants playing on their own instrument and with their normally used ergonomic equipment, thereby supporting the weight of the instrument as usual but remaining in the standardized body position as required for the previously measured violin positions A1 through B2 (Table 2 and Figure 2).
- Biomechanical data were collected in our university section’s hand laboratory (see section 3.4.3, as well as https://www.zzm.ch) (Zurich Centre for Musicians’ Hands, 2022).

**Table 1:** Laboratory instruments used in tests.

**Instrument** Description in brackets: First body dimension, then string length 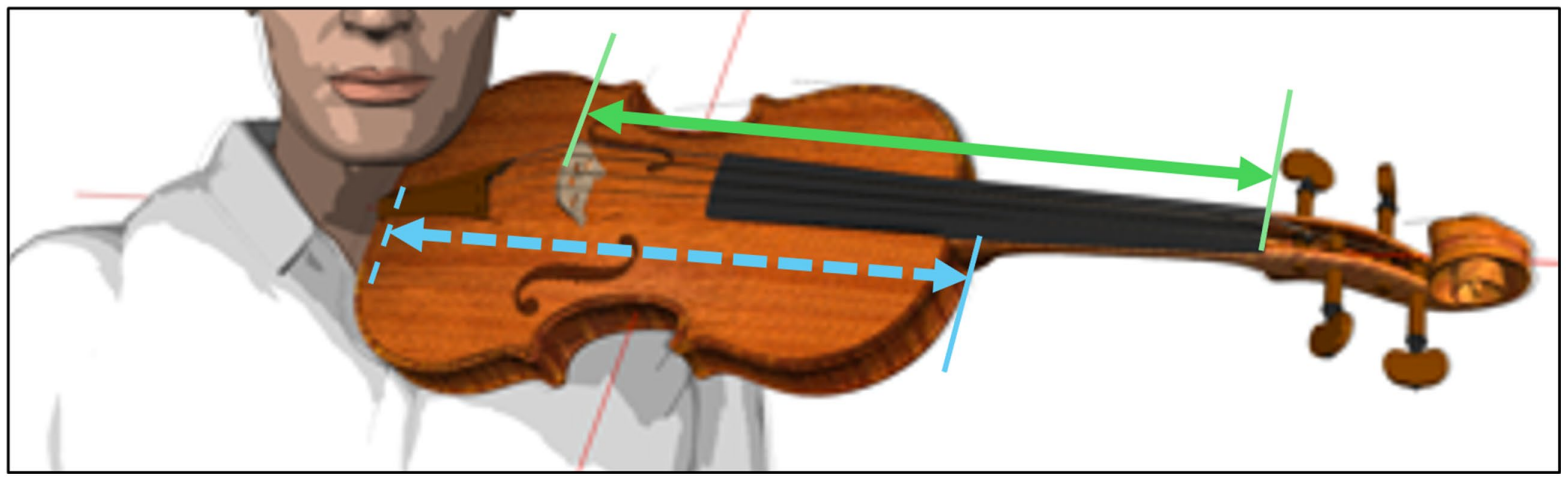	**Specification of instrument body length [mm, blue arrow]**	**Specification of string length [mm, green arrow]**
Viola 1 (small/short)	405	365
Viola 4 (large/long)	420	380

**Table 2:** Standardized instrument positions tested for comparison.

**Position**	**Description in the language of the hand laboratory**
A1: LoAx-CSP = 20°, LatAx = 20° 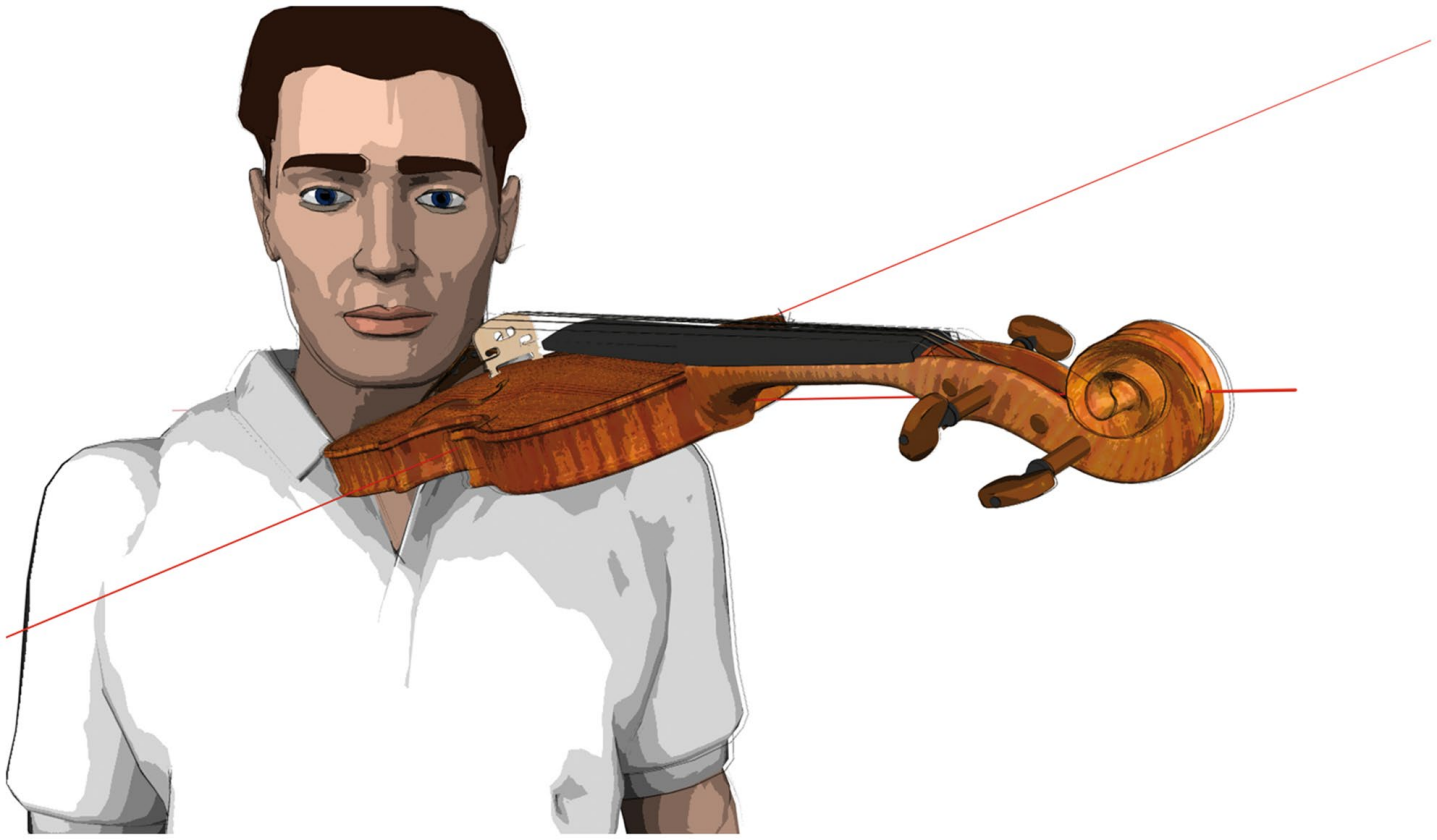	Longitudinal, central instrument body axis points toward the left lamina of thyroid cartilage (vertical *via* clavicular insertion of left sternocleidomastoid muscle), at a 20° angle to the sagittal axis, lateral central instrument body axis points 20° forwards/downwards.
A2: LoAx-CSP = 20°, LatAx = 50° 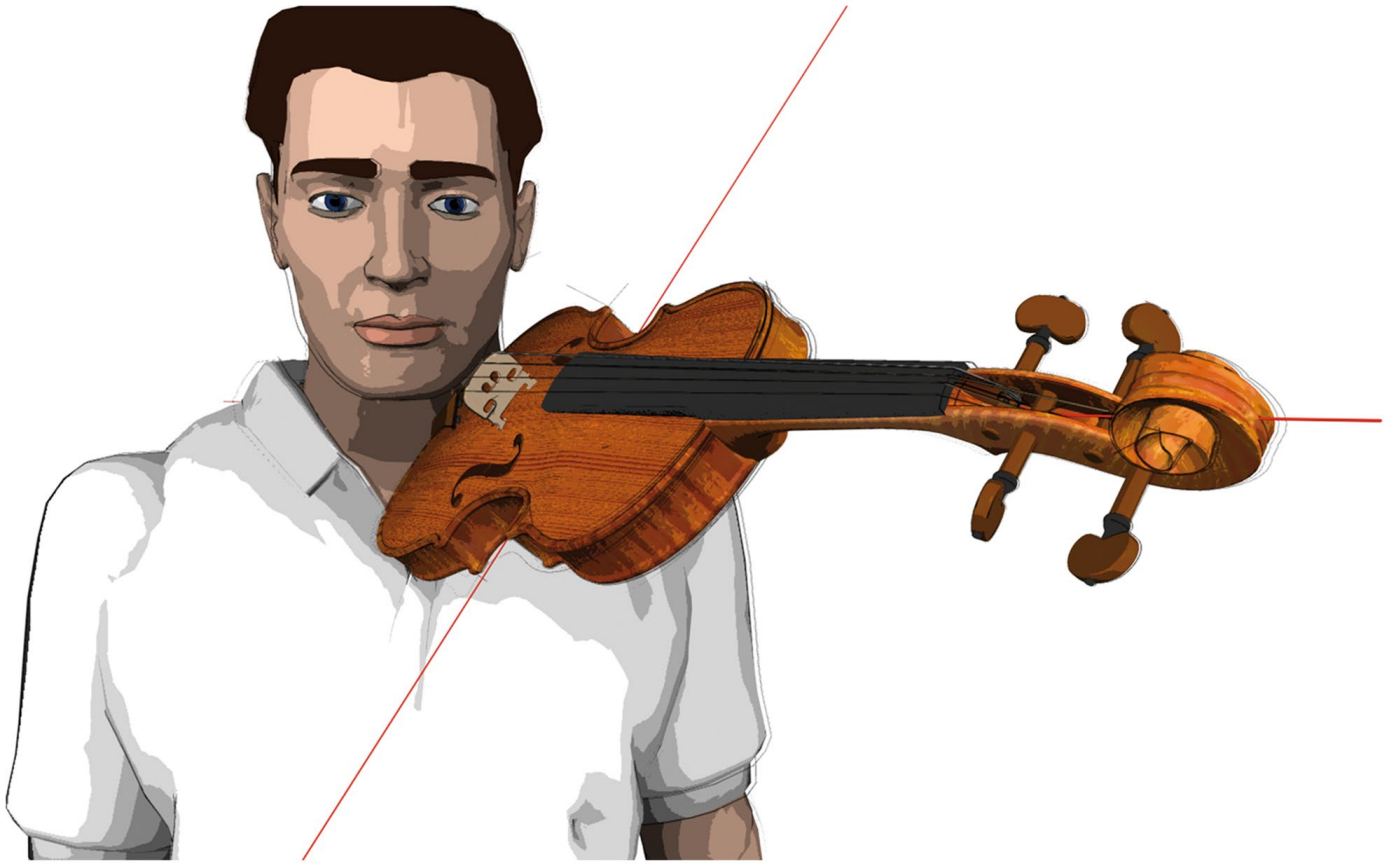	Longitudinal central instrument body axis points toward the left lamina of thyroid cartilage (vertical *via* the clavicular insertion of the left sternocleidomastoid muscle), at a 20° angle to the sagittal axis, lateral central instrument body axis points 50° forwards/downwards.
B1: LoAx-CSP = 50°, LatAx = 20° 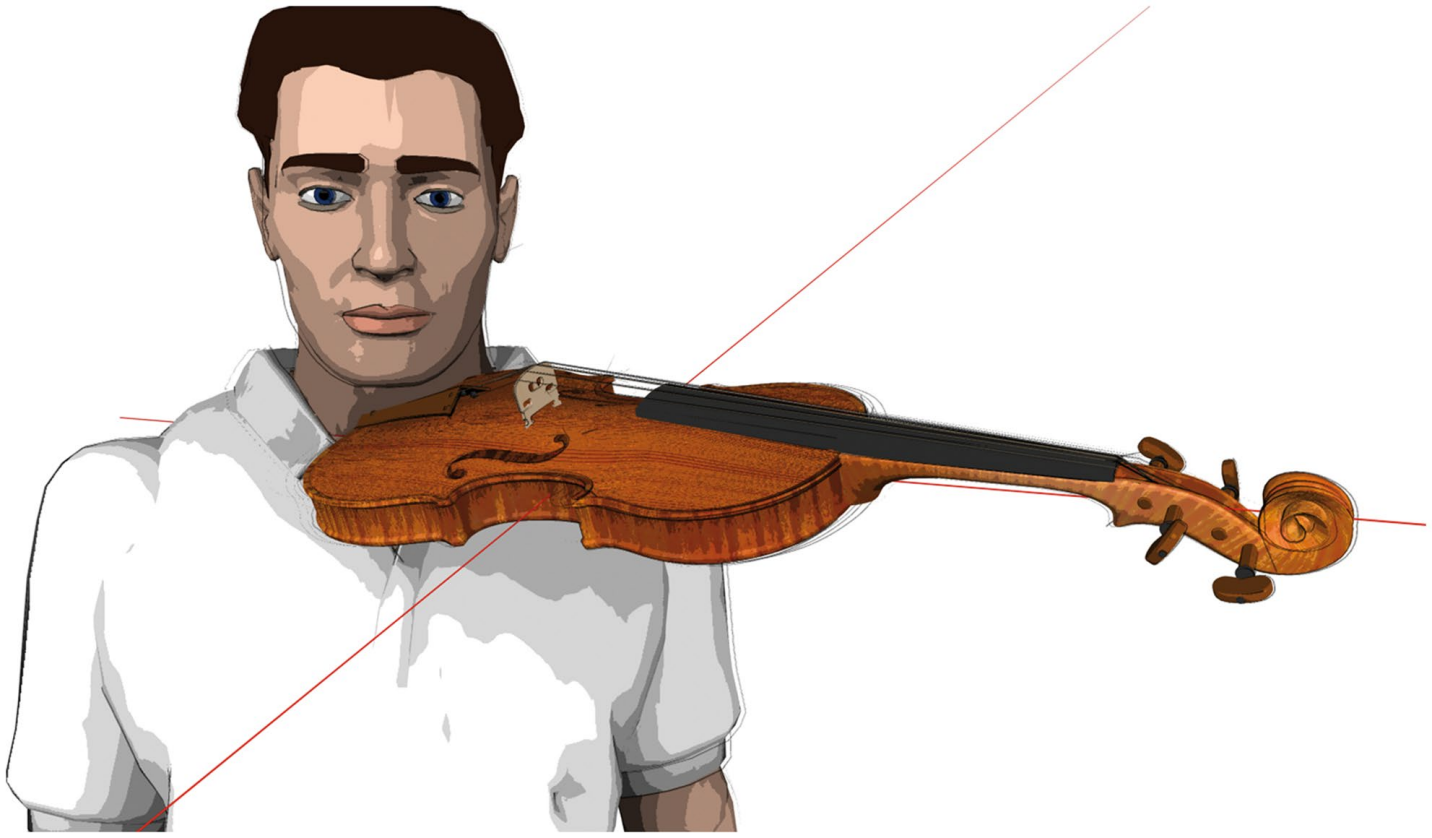	Extension of longitudinal central instrument body runs to clavicular insertion of the right sternocleidomastoid muscle at an angle of 50° to the sagittal axis, lateral central instrument body axis points 20° forwards/downwards.
B2: LoAx-CSP = 50°, LatAx = 50° 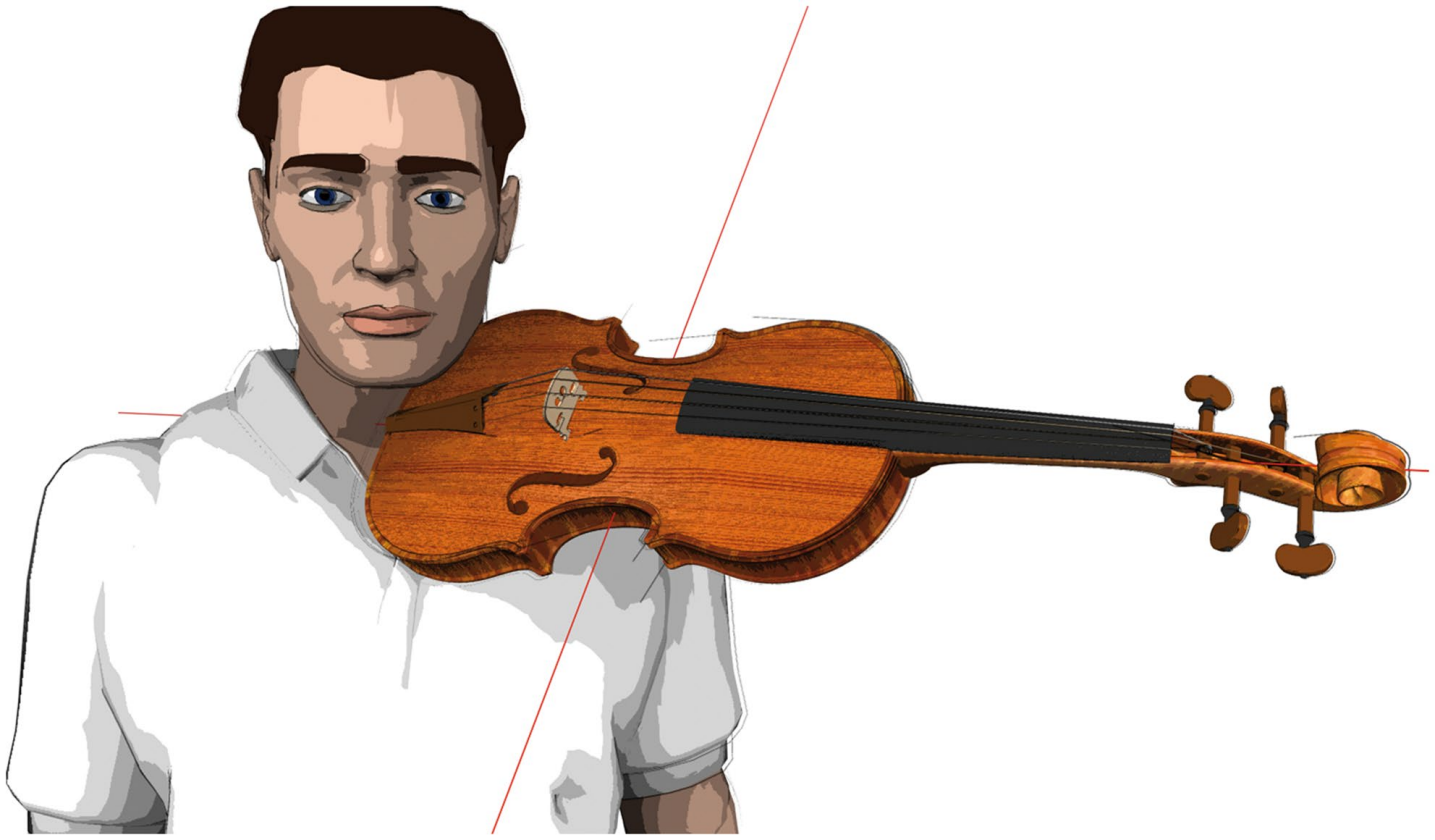	Extension of longitudinal central instrument body runs to clavicular insertion of the right sternocleidomastoid muscle at an angle of 50° to the sagittal axis, lateral central instrument body axis points 50° forwards/downwards.

### 3.3 Technical prerequisites

To ensure measurements under standardized conditions (see Table 2), our team drew upon pre-existing technical infrastructure from previous studies (Hildebrandt et al., 2021; Margulies et al., 2023), in particular the use of a device allowing three-dimensional fitting of the laboratory instruments to the player with precisely reproducible heights and angles. The use of this device allows for the minimization of excess holding work in the head, neck, shoulder, arm, and hand while playing an instrument, as the player is not reliant on the use of ergonomic aids. This aims to reduce/eliminate confounding variables such as individual playing and postural habits or interaction effects between an instrument fitted with ergonomic equipment and the player. This methodology allowed for intraindividual comparison between all violas and their positions tested. The standardized instrument positions were defined by estimates based on a range of recommendations of internationally renowned players’ and teachers’ instrument positions and considered typical in teaching traditions of the last few centuries (Flesch, 1929/1978; Galamian, 1983; Rônez-Kubitschek, 2012; Rostal, 1993).

The laboratory instruments had the dimensions listed in Table 1. The instruments were used without ergonomic equipment (i.e., chin-rest or shoulder pad) as they were fitted in the fixation device, as schematically depicted in Figure 2. String tension was specified by tuning the instrument to concert pitch 442 Hz.

### 3.4 Target parameters

#### 3.4.1 Electromyography (EMG)

EMG data for the four muscles listed in Table 3 were collected in the four standardized instrument positions, as well as the violist’s own position normally used and with their own instrument. The muscles focused on are representatives of a chain of muscles used when playing the violin and the viola (Ackermann and Adams, 2003; Obata and Kinoshita, 2012; Park et al., 2012; Shan and Visentin, 2003; Steinmetz et al., 2016) They are accessible for surface EMG measurement (Konrad, 2011) and are representative for playing motions of the fingers, wrist, supination, and compensation movements of the shoulder girdle, especially in higher playing positions. Electrode positions were documented photographically and remained in place throughout the entire measurement phase. For measurements, a 4-channel Nexus-10 Mark-II device from Mind Media B.V. (Herten, The Netherlands) was used. EMG data were filtered with a 3rd-degree Butterworth filter with a frequency range of 100 to 500 Hz to remove artefacts (e.g., due to electrical heart activity). In addition to gathering data on muscle activation for each of the four muscles singly, the sum of values for all four muscles was used as a composite measure for muscle activation while playing in order to compare EMG values with overall subjective effort values on the BORG scale. Average values of the derived peak amplitude values (RMS, root mean square) and peaks per time (TURNS, number of positive and negative peaks per time) were calculated for the defined time intervals (Konrad, 2011). In order to enable interindividual comparability of EMG data, measures were expressed as a percentage of those at maximum voluntary contraction (MVC). MVC was determined as the average of three maximal contractions in a standardized position prior to the test. Study participants were asked to assume standardized body positions for MVC measurement (Konrad, 2011) and challenged to carry out three maximal voluntary contractions for each of the four muscles (“*Upon my command, build up as high a resistance to my grip as you can—NOW!*”). MVCs were monitored throughout to ensure maximal effort was being put in by the participants.

**Table 3:** EMG electrode positioning.

**Muscle**	**Electrode position**
(a) Biceps brachii	1st electrode 2 subject’s finger widths (fw)* distal of deltoid punctum 2nd electrode 2 cm distal to 1st electrode *2 subject’s fw = index finger and middle finger
(b) Extensor digitorum communis	1st electrode 3 subject’s fw** distal of lateral epicondyle 2nd electrode 2 cm distal to the 1st electrode **3 subjects’ fw = index finger, middle finger, and ring finger
(c) Flexor carpi ulnaris	1st electrode 3 subject’s fw^†^ distal of medial epicondyle 2nd electrode 2 cm distal to 1st electrode ^†^ 3 subjects’ fw = index finger, middle finger, and ring finger
(d) Pectoralis major	1st electrode 2 subject’s fw^‡^ lateral of sternal head base 2nd electrode 2 cm lateral to 1st electrode ^‡^ 2 subject’s fw = index finger and middle finger

#### 3.4.2 Subjectively felt effort (BORG)

After playing the predefined tune in each of the four positions (Table 2), subjectively perceived effort in the left hand, arm, and shoulder was specified by the study participants by using the Borg scale, a tried-and-tested self-assessment scale used in sports medicine and performance diagnostics for recording subjectively perceived effort (Borg, 1982). The scale ranges from 6 (least effort, ‘no exertion at all’) to 20 (highest degree of effort, ‘maximal exertion’) (e.g., 13 = ‘somewhat hard’). The question put forward to the study participants after playing was: “How would you describe your overall effort in the left hand and arm when playing the tune by means of a value on this effort scale? For the overall tune,” Borg’s overall effort perception was documented in view of juxtaposing these results with aggregated EMG values. Reporting BORG effort values singly for the four muscles proved too challenging and was therefore not included.

#### 3.4.3 Biomechanical parameters (collected by means of the BHM)

The parameters, as shown in Table 4, were recorded by means of the Biomechanical Hand Measurement (BHM) according to Wagner (2005):

**Table 4:** Biomechanical parameters recorded.

**Parameter group 1**	**Parameter group 2**	**Parameter group 3**	**Parameter group 4**
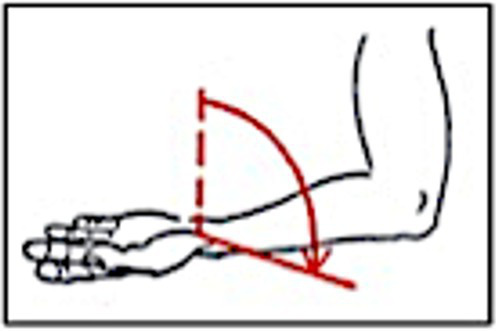 Active and passive supination	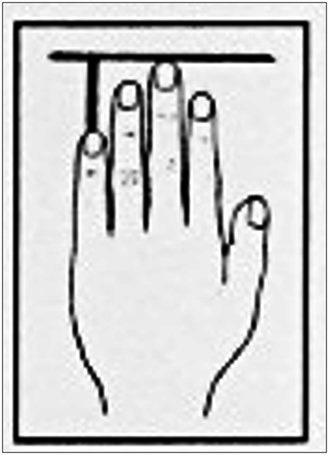 Difference in finger length between the 3rd and 5th fingers	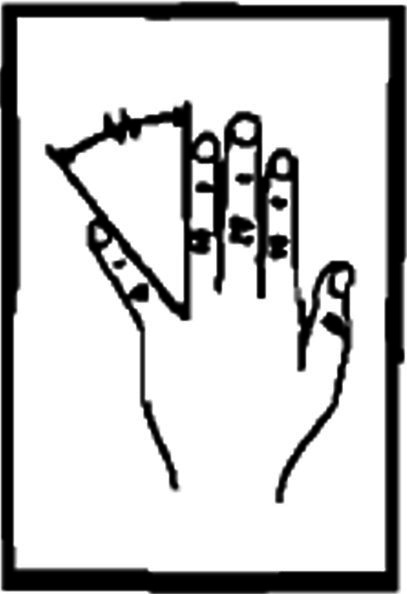 Passive spreading of fingers 2–5 (illustration inserted as an example for spreading of fingers 4–5)	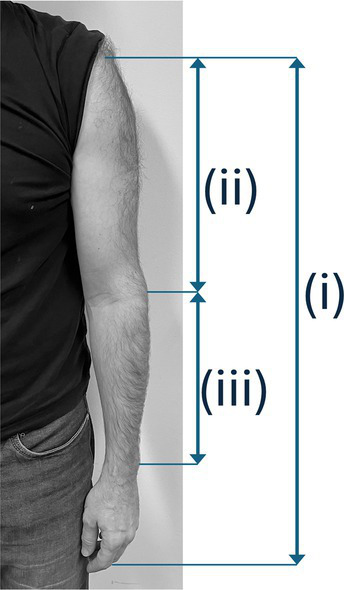 Overall arm length and length of upper and lower arm

(1) Passive supination ability of the left lower arm at torque levels 16 Ncm (or 250 g weight) and 30 Ncm (or 500 g weight): Indicates effort or ease of the hand reaching basic positions on the viola (Parameter group 1). Measurements result in an angle degree describing the deviation of the left forearm, hand, and wrist from the neutral position. (2) Difference in finger length between the left hand’s 3rd and 5th fingers: Indicates the 5th finger’s range on the viola and the need for compensation by supination. Measurements result in a millimeter value (Parameter group 2). (3) Passive spreading of fingers 2–5: Indicates the individual range of finger movements on the viola’s string and the need for compensation by other biomechanical parameters observed. Measurements result in an angle degree value (Parameter group 3). In addition, (4) the overall length of the arm (i = sub-acromial space to the middle finger’s tip), upper arm singly (ii = sub-acromal space to epicondylus lateralis humeri), lower arm singly (iii = epicondylus lateralis humeri to processus styloideus ulnaris) (Parameter group 4) were newly added as part of the measurement process, as they were expected to influence muscle activation and perceived effort in interdependency with the viola’s position as well as dimension and string length (cf. Table 1).

### 3.5 Study participant positioning

The participants positioned themselves on the instrument fitting device’s platform (see Figure 2), upright, head position looking straight ahead at the music stand with the tune placed on it. Individually adjustable stabilizers on both sides of the study participants’ heads ensured minimization of strain on the neck and shoulder muscles during measurements. Participants were instructed to choose a shoulder position that felt as relaxed as possible before going into the playing position. The positions of feet and knees were defined, marked, and positioned to ensure a comfortable standing position throughout measurement phases. Study participants were asked to remain in the same body position during measurements to permit accurate intraindividual data comparisons. The body position was monitored and documented with two video cameras (frontal and from the side).

### 3.6 Measurement procedure

The measurement order for each of the four standardized viola positions A1 to B2 (see Table 2), and the participants’ playing position with his/her own instrument (“Free”) were randomized and included the following steps:

- Within a 10-s countdown, the participant went into playing position with the left hand.
- The fingers were positioned over the notes specified by the tune.
- The participant played the tune (Figure 1) without a bow and then let the left hand sink back down into the starting position.
- During a break, the study participant rested the hand and arm without self-massage, tapping, or shaking the hand.

This procedure was applied when playing the tune both in the 2nd position and the 6th position. These positions differ from each other: when in the second position, the player’s hand is located further away from the player’s body, with less need for compensation movements of the hand and arm to reach the required notes; in the sixth position, the player’s hand is nearer to the player’s body, with more need for compensation movements.

### 3.7 Statistical procedures and analysis

Data assessment was carried out using SPSS 23ff for Windows and R 4.5. The three conventional significance levels were used for all calculations: “significant”: *p* < 0.05 (*), “highly significant”: *p* < 0.01 (*), “very highly significant”: *p* < 0.001 (***), which are each non-directional, i.e., two-tailed. Correlations between biomechanical data and relevant findings on muscle activity and perceived effort using the two test instruments in the respective instrument and playing positions as well as when study participants use their own viola was analyzed using univariate and multivariate statistical procedures (in particular: variance analysis (ANOVA), multiple comparison of means including the Scheffé test for homogeneous subgroups, parametric or non-parametric test procedures depending on the data level). In the multilevel regression analysis of instrument position (A1, A2, B1, B2, and Free) on EMG and BORG, within-subject repeated measures for instrument dimension (V1, V4, and Free) and hand playing position (6 L and 2 L) were taken into account as 2^nd^ level and participant as 3rd level (random effects).

## 4 Results

### 4.1 Description of the sample, summary statistics

The sample consisted of 31 violists, 15 men and 16 women. Their mean age was 24.8 years (SD = 3.6) with a minimum of 18 and a maximum of 33 years. The unit of analysis for muscle activation (EMG) and subjectively perceived effort (BORG) is *N* = 558 observations, deriving from 31 persons with 18 measurement points per person. The 18 measurement points consisted of tests in four standardized instrument positions (A1, A2, B1, and B2, cf. Table 2) for each of the two laboratory instruments (V1 and V4, cf. Table 1) as well as the participants’ own instrument (Free), in which each participant was measured two times playing a predefined, 16-s tune (cf. Figure 1) in the 6th and the 2nd hand playing position (6 L and 2 L) in randomized order (2*4*2 + 1*2 = 18). No observations were missing.

Muscle activation was measured over four channels (cf. Table 3) and expressed as a percentage of MVC over time (sample rate: 2′048 measurement points per second = 32′768 measurement points in a total collapsed to mean values per person). The sum of the four channels forms the combined EMG scale (EMG Overall), which we used to measure global muscle activation levels. High values in EMG Overall indicate high levels of muscle activation. The BORG scale was used to measure subjectively perceived effort, whereby this standardized scale ranges from value 6 (least effort) to 20 (highest degree of effort). Borg scale feedback yielded one measurement point per performance of tune = 558 measurement points in total for a 16-s tune overall. Biomechanical parameters were recorded by means of the Biomechanical Hand Measurement (BHM) according to Wagner (2005). The units for the parameters in focus are expressed either in degrees, centimeters, or millimeters, and per study participant, measurement results in one value per parameter observed (hence *N* = 31 per parameter).

The summary statistics for the sample are listed in Table 5.

**Table 5:** Summary statistics of parameters under test.

**Parameter (left hand)**	***N***	**Min**	**Max**	**Mean**	**SD**
EMG and BORG					
EMG Overall (all four muscles aggregated)	558	0.10	15.24	3.99	2.20
BORG Overall (hand, arm, and shoulder expressed as one score)	558	6.00	19.00	10.43	2.99
Biomechanics					
Overall_Arm_Length (cm)	31	65.5	83.5	74.03	4.49
Upper_Arm_Length (cm)	31	26	34	30.16	1.74
Lower_Arm_Length (cm)	31	21	28	25.08	1.73
Finger_Length_Diff_3_5 (mm)	31	33	46	38.48	3.69
Supination_Active (°)	31	78	111	93.87	8.39
Supination_Passive_500_1 (°)	31	55	112	91.00	13.98
Supination_Passive_250 (°)	31	19	104	66.71	26.56
Supination_Passive_500_2 (°)	31	53	112	90.94	14.28
Supination_Passive_750 (°)	31	75	121	101.10	12.03
Supination_Passive_1000 (°)	31	87	127	106.94	11.38
Thumb_Abd_Passive (°)	31	34	73	53.39	8.95
Finger_Spreading_Passive_1_3 (°)	31	21	49	32.13	6.50
Finger_Spreading_Passive_1_4 (°)	31	59	109	79.61	12.25
Finger_Spreading_Passive_1_5 (°)	31	66	126	95.19	14.51
Fingers_Spreading_Passive_2_3 (°)	31	23	62	41.68	8.46
Fingers_Spreading_Passive_3_4 (°)	31	17	41	30.10	6.27
Fingers_Spreading_Passive_4_5 (°)	31	24	67	45.68	9.24
Fingers_Spreading_Passive_2_4 (°)	31	34	83	54.48	10.70
Fingers_Spreading_Passive_3_5 (°)	31	35	80	60.48	11.22
Fingers_Spreading_Passive_2_5 (°)	31	40	100	70.06	13.23

### 4.2 Main hypotheses 1, pertaining to the instrument’s dimensions

The main hypothesis 1, “The larger the instrument’s dimensions and its string length, the higher muscle activity and perceived effort in the violist’s left arm will be present when playing,” was not confirmed for Overall EMG (all four EMG channels, instruments, and hand positions aggregated, cf. 4.2.1). For subjectively perceived effort (BORG), the main hypothesis 1 was confirmed (cf. 4.2.2).

#### 4.2.1 Results for muscle activation (EMG)

A comparison between the three tested instruments, V1, V4, and Free (collapsed for all instrument and hand positions), shows the highest mean values for aggregated %MVC over four EMG channels for V4 (*N* = 248, mean = 3.59, SD = 1.82), followed by instrument V1 (*N* = 248, mean = 3.39, SD = 1.76) and then Free (*N* = 62, mean = 3.25, SD = 1.73). ANOVA analyzing this total instrument effect on Overall EMG shows no statistically significant result [*F*(2, 555) = 1.291, *p* = 0.276]. A multiple comparison of means (Scheffé), comparing each of the three groups to the other two one by one, shows that none of the three groups differ significantly from the others.

At the level of single EMG channels, the same comparison for the *pectoralis major* muscle shows the highest mean values for aggregated %MVC for V4 (*N* = 248, mean = 3.16, SD = 2.20), followed by instrument V1 (*N* = 248, mean = 3.00, SD = 2.15) and then Free (*N* = 62, mean = 2.27, SD = 1.69). These differences are statistically significant in the analysis of variance: [*F*(2, 555) = 4.526, *p* = 0.013]. A multiple comparison of means (Scheffé) comparing each of the three groups to the other two one by one, shows that the main differences occur between instrument V4 and instrument Free (difference = 0.89, *p* < 0.05) and between V1 and Free (difference = 0.74, *p* = 0.051, trend), while the difference between instrument V4 and V1 is not significant (difference = 0.15, *p* = 0.723). For the other muscles under test, multiple comparisons of means yielded no significant differences for the 16-s tune. For further reference, please see Supplementary Table S2.

#### 4.2.2 Results for subjectively perceived effort (BORG)

**Figure 3:**
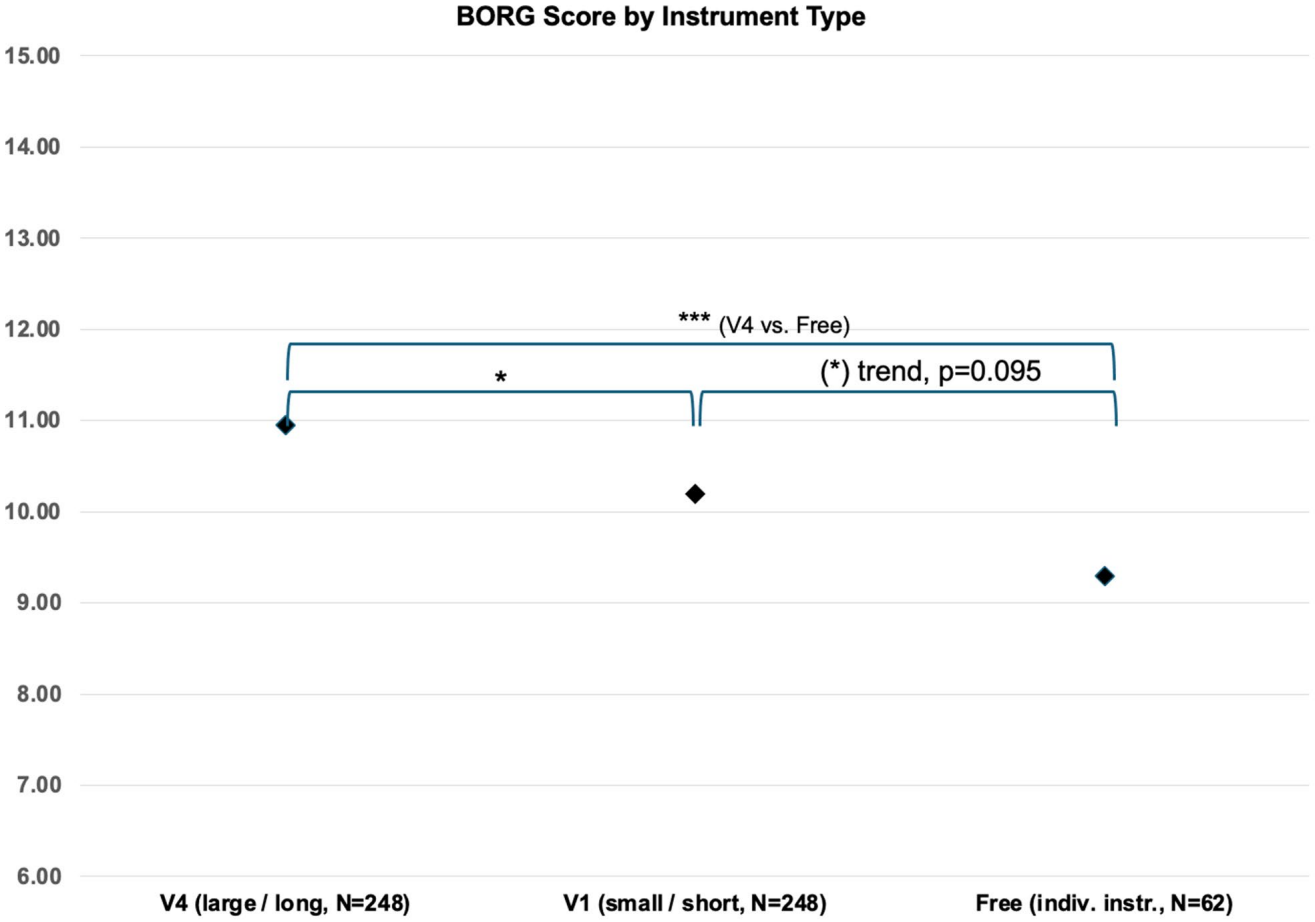
Borg scale value comparison by instrument under test.

A comparison between the three tested instruments, V1, V4, and Free (again collapsed for all instruments and hand positions), shows the highest mean BORG Overall values for instrument V4 (*N* = 248, mean = 10.95, SD = 3.12) followed by instrument V1 (*N* = 248, mean = 10.20, SD = 2.875) and then Free (*N* = 62, mean = 9.29, SD = 2.42) (see Figure 3). These differences are highly significant in the ANOVA: [*F*(2, 555) = 9.266, *p* < 0.05]. A multiple comparison of means for the three instruments (Scheffé) shows that mean values for BORG Overall differ significantly between instrument V4 and Free (difference = 1.66, *p* < 0.001) and significantly between V4 and V1 (difference = 0.75, *p* < 0.05), while there is a trend to a difference between V1 and Free (difference = 0.91, *p* = 0.095). Therefore, for subjectively perceived effort (BORG), main hypothesis 1 was confirmed for subjectively perceived effort.

### 4.3 Results pertaining to instrument positioning effects

Sub-hypothesis 1, “The more the longitudinal axis of the instrument is pointing toward the front (relative to the sagittal plane) and the more horizontal the lateral axis of the instrument (relative to the horizontal plane), the more muscle activation and perceived effort will be present in the violist’s left arm when playing,” was confirmed for both muscle activation (EMG) and perceived effort (BORG).

#### 4.3.1 Results for muscle activation (EMG)

**Figure 4:**
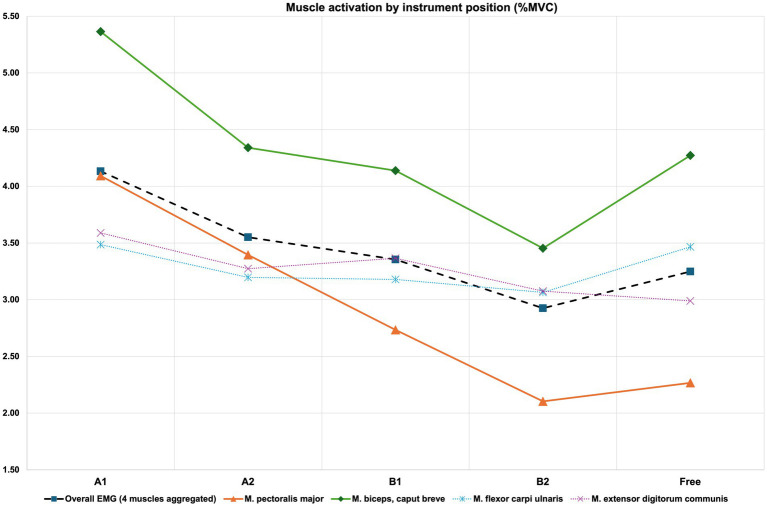
Muscle activation by instrument position for aggregated EMG (“Overall”) and single-muscle level (%MVC). For SE and significance levels, please refer to Table 6 below.

The following results for the standardized instrument positions A1, A2, B1, and B2 show data collapsed for both laboratory instruments (V1 and V4) and hand positions (2nd and 6th playing position) played. For aggregated EMG data as well as at the single-muscle level for the *pectoralis major* and *biceps brachii caput breve* muscle, results for EMG show a gradation in muscle activation with the A1 viola position yielding the highest values, followed by instrument positions A2, B1, and B2, as the instrument position linked to the lowest levels of muscle activation. Instrument position “Free” (i.e., the study participants’ viola played with their usual ergonomic set-up shows an increase of muscle activation) (see Figure 4 below). For a detailed overview of the results, please refer to Supplementary Tables S3a–c.

The differences between the five instrument positions are highly significant in the ANOVA: [*F*(4, 553) = 7.953, *p* < 0.001]. A one-by-one multiple comparison of the means for the five instrument positions under test (Scheffé) shows that mean values for EMG Overall differ significantly between instrument position A1 and all other positions except for position A2. For details, please refer to Supplementary Tables S3a–c.

At the level of single EMG channels, the same comparison for the *pectoralis major* muscle shows highest mean values for aggregated %MVC for A1 (*N* = 124, mean = 4.09, SD = 2.56), followed by A2 (*N* = 124, mean = 3.40, SD = 2.04), B1 (*N* = 124, mean = 2.73, SD = 1.95), Free (*N* = 62, mean = 2.27, SD = 1.69), and finally B2 (*N* = 124, mean = 2.10, SD = 1.50). These differences are statistically significant in the analysis of variance: [*F*(4, 553) = 19.043, *p* < 0.001]. A multiple comparison of means (Scheffé) comparing each of the five instrument positions to the other two one by one for this specific EMG channel, shows that EMG activation levels differ significantly between each other (*p* < 0.001) except between instrument position A1 and A2 (n.s.).

For the *biceps brachii muscle*, a similar pattern arises: Results show highest mean values for aggregated %MVC for A1 (N = 124, mean = 5.37, SD = 3.67), followed by A2 (N = 124, mean = 4.34, SD = 2.62), B1 (N = 124, mean = 4.14, SD = 2.70), Free (N = 62, mean = 4.27, SD = 3.24), and finally B2 (N = 124, mean = 3.45, SD = 2.31). These differences are statistically significant in the analysis of variance: F(4, 553 = [6.852], p < 0.001). A multiple comparison of means (Scheffé) comparing each of the five instrument positions to the other two one by one for this specific EMG channel, shows that EMG activation levels differ significantly between A1 and B2 (difference = 1.91, *p* < 0.001) and significantly between A1 and B1 (difference = 1.23, p < 0.05), but are non-significant in the other comparisons. For further details, please refer to Supplementary Tables S3a–c.

For the remaining two muscles (*musculus flexor carpi ulnaris and extensor digitorum communis*), no significant interaction effects between instrument positioning and muscle activation were found.

In Table 6, the results of a multilevel linear regression model of instrument position (A1, A2, B1, B2, and Free; 1st level) on muscle activation (EMG) are presented. Instrument dimension (V1, V4, and Free) and hand playing position (6 L and 2 L) are taken into account as repeated measures at the 2nd level and participant as the 3rd level. As shown, instrument position A1 leads to the highest muscle activation of all instrument positions, followed by positions A2, B1, Free, and B2, respectively. All positions show significantly lower muscle activation compared to A1. The determination coefficient R^2^ of the model is 0.054; thus, 5.3% of muscle activation variance is explained by the positions.

**Table 6:** Regression analysis for the viola position to EMG.

**Position**	***b***	**se**	***z***	***p***		
VIP A1	0.00				Number of obs	558
VIP A2	−0.58	0.06	−10.09	<0.001		
VIP B1	−0.78	0.09	−12.61	<0.001		
VIP B2	−1.21	0.09	−13.12	<0.001	Marginal *R*-squared	0.053
VIP Free	−0.88	0.15	−6.04	<0.001		
Constant	4.13	0.28	14.86	<0.001	RMSE	0.367

#### 4.3.2 Results for subjectively perceived effort (BORG)

**Figure 5:**
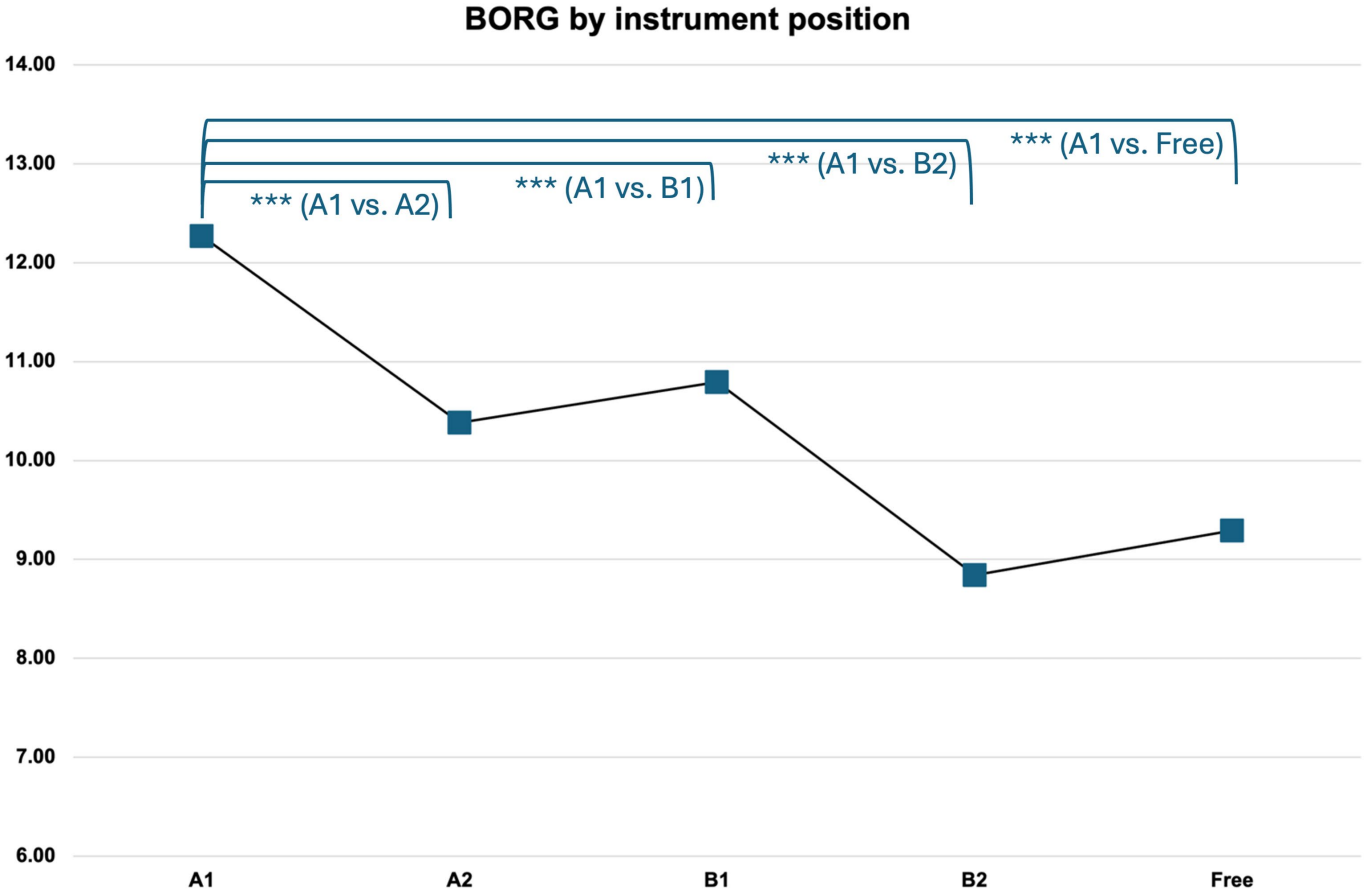
BORG effort levels by instrument position. For SE and significance levels, please refer to Table 7 below.

For BORG data, collapsed for both laboratory instruments (V1 and V4) and hand positions (2^nd^ and 6th playing position) played as above for EMG, results also show a gradation with a pattern comparable to EMG (see 4.3.1 above). As for EMG, effort levels are highest for the A1 viola position, followed by A2, B1, and B2, as the instrument position linked to the lowest levels of subjectively perceived effort. Instrument position “Free” sees a slight increase relative to B2 (see Figure 5 below). For a detailed overview of the results, please refer to Supplementary Tables S4a–c.

The differences between the instrument positions are highly significant in the ANOVA: [*F*(4, 553) = 27.747, *p* < 0.001]. A multiple comparison of means for the five instrument positions under test (Scheffé) shows that mean values for EMG Overall differ significantly (p < 0.001) between instrument position A1 and each of the other positions. For details, please refer to Supplementary Tables S4a–c.

Table 7 shows a comparable pattern in the results for the multilevel linear regression model of instrument position on the subjectively perceived effort (BORG-scale) as for EMG above (Table 6). B2 and Free were perceived as the least demanding, A2 and B1 demanded less effort compared to A1, with the most perceived effort. Compared to the reference position A1, again, all other positions show significantly lower values. Marginal R^2^ of the model is 0.169, thus about 17% of subjectively perceived effort variance is explained by the positions.

**Table 7:** Regression analysis for viola position to BORG.

**Position**	***b***	**se**	***Z***	***p***		
VIP A1	0.00				Number of obs	558
VIP A2	−1.89	0.23	−8.33	<0.001		
VIP B1	−1.48	0.21	−6.99	<0.001		
VIP B2	−3.43	0.25	−13.91	<0.001	Marginal R-squared	0.169
VIP Free	−2.98	0.33	−8.98	<0.001		
Constant	12.27	0.34	36.61	<0.001	RMSE	1.180

#### 4.3.3 Sub-analysis: instrument comparison V1 in A1 vs. V4 in B2

In view of deepening our understanding for the relationship between an instrument’s dimension and its positioning effects on the target parameters, a sub-analysis was carried out comparing instrument V1 (small dimension, short string length) in position A1 (hypothesized as the instrument position linked to highest degrees of muscle activation and subjectively perceived effort) with instrument V4 (large dimension, long string length) in position B2 (hypothesized as the instrument position linked to lowest degrees of muscle activation and subjectively perceived effort).

##### 4.3.3.1 Results for muscle activation (EMG)

**Figure 6:**
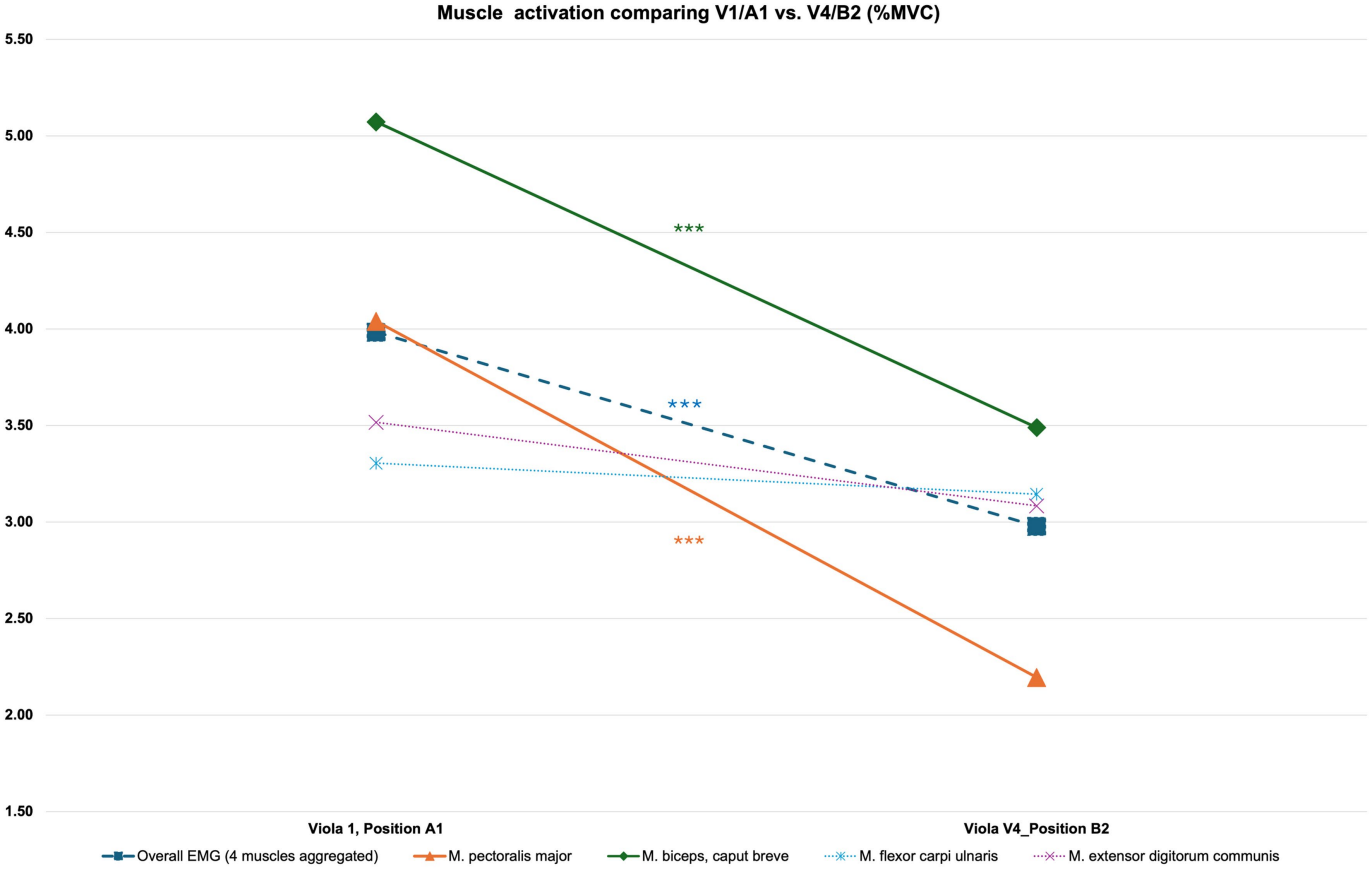
Muscle activation comparison V1/A1 vs. V4/B2 for aggregated EMG and at the single-muscle level. For further details, please refer to Supplementary Tables S5a–e.

A comparison between laboratory instrument V1 in position A1 with laboratory instrument V4 in position B2 shows higher mean values for aggregated %MVC over four EMG channels for the first testing condition compared to the second: The %MVC for Instrument V1 in position A1 (N = 62, mean = 3.98, SD = 1.94) is higher than the value for the same parameter in testing condition Instrument V4 in position B2 (N = 62, mean = 2.98, SD = 1.46). ANOVA for the instrument dimension and positioning effect on Overall EMG shows a statistically highly significant result [*F*(8, 549) = 4.191, *p* < 0.001]. A multiple comparison of means (Scheffé) comparing the different instrument dimensions with their positioning one by one shows no significant results at the highest aggregation level for the four muscles under test: none of the three groups differ significantly from another (see Figure 6).

Comparing the two aforementioned test positions at the level of single EMG channels for the *pectoralis major* muscle, the %MVC for Instrument V1 in position A1 (*N* = 62, mean = 4.04, SD = 2.55) is again higher than the value for the same parameter in testing condition Instrument V4 in position B2 (N = 62, mean = 2.19, SD = 1.56). ANOVA for the instrument dimension and positioning effect on Overall EMG shows a statistically highly significant result [*F*(8, 549) = 4.191, *p* < 0.001]. A multiple comparison of means (Scheffé) comparing the different instrument dimensions and positioning combined one by one, shows highly significant results for this muscle (difference = 1.84, *p* = 0.001). For details, please refer to Supplementary Tables S5a–e.

Comparing the two aforementioned test positions at the level of single EMG channels for the *biceps brachii, caput breve* muscle, the %MVC for Instrument V1 in position A1 (*N* = 62, mean = 5.07, SD = 3.35) is again higher than the value for the same parameter in testing condition Instrument V4 in position B2 (*N* = 62, mean = 3.49, SD = 2.27). ANOVA for the instrument dimension and positioning effect on Overall EMG shows a statistically highly significant result [*F*(8, 549) = 3.661, *p* < 0.001]. A multiple comparison of means (Scheffé) comparing the different instrument dimensions and positioning combined one by one, shows no significant results for this muscle.

For the remaining two muscles (*musculus flexor carpi ulnaris and extensor digitorum communis*), no significant interaction effects between instrument positioning and muscle activation were found.

##### 4.3.3.2 Results for subjectively perceived effort (BORG)

**Figure 7:**
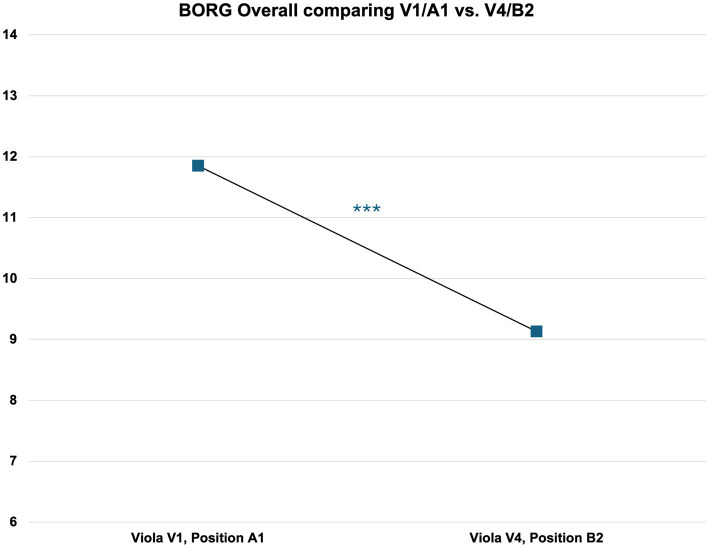
Differences in perceived effort levels (BORG) between V1/A1 and V4/B2. For further details, please refer to Supplementary Tables S6a–c.

A comparison between laboratory instrument V1 in position A1 with laboratory instrument V4 in position B2 shows higher mean values for BORG for the first testing condition compared to the second: BORG values for Instrument V1 in position A1 (*N* = 62, mean = 11.85, SD = 3.062) is higher than the value for the same parameter in testing condition Instrument V4 in position B2 (*N* = 62, mean = 9.13, SD = 2.320) (Figure 7).

ANOVA for the instrument dimension and positioning effect on BORG shows a statistically highly significant result [*F*(8, 549) = 15.307, *p* < 0.001]. A multiple comparison of means (Scheffé) comparing the different instrument dimensions with their positioning one by one shows highly significant results in this specific comparison (difference = 2.718, *p* < 0.001). For details, please refer to Supplementary Tables S6a–c.

### 4.4 Results pertaining to biomechanical parameters

#### 4.4.1 Results for active and passive supination

Hypothesis 2, “The lower the active and passive supination ability, the more muscle activation and perceived effort will be present in the violist’s left hand and arm when playing,” was partly confirmed at the single-muscle level.

The linear relationship between Active Supination, Passive Supination, and EMG Overall and Subjectively Perceived Effort (BORG Overall) was assessed by computing Pearson’s *r* correlation coefficient for all three instruments (V1, V4, Free), instrument positions (A1, A2, B1, B2, Free), and hand playing positions (2 L and 6 L) collapsed.

Correlation analysis for supination ability (active as well as passive) and EMG Overall did not yield significant results for the entire group. However, for correlation analysis between the same parameters and single EMG Channel C (M. flexor carpi ulnaris), a negative correlation is reported for parameter Supination_Active (r = −0.106, *p* < 0.05). Further negative correlations were found between parameters Supination_Passive_250 and EMG Channel C (r = −0.283, p < 0.001), Supination_Passive_500 and EMG Channel C (r = −0.341, p < 0.001), Supination_Passive_750 and EMG Channel C (r = −0.272, p < 0.001), and Supination_Passive_1000 and EMG Channel C (r = −0.188, p < 0.001).

Between Supination_Passive_250 and subjectively perceived effort (BORG Overall, i.e., for the entire upper limb and shoulder), a negative correlation is reported (r = 0.106, p < 0.05).

#### 4.4.2 Results for further biomechanical parameters

Sub-Hypothesis 2, “The shorter the overall length of the arm and its parts, the larger the difference in length between the little finger and the middle finger, the lower values for passive spreading between fingers, the more muscle activity and perceived effort will be present in the violist’s left arm when playing,” was partly confirmed.

Negative correlations between arm parameters and Overall EMG were found for Overall_Arm_Length (*r* = −0.359, *p* < 0.001), Upper_Arm_Length (*r* = −0.368, *p* < 0.001), and Lower_Arm_Length (*r* = −0.328, *p* < 0.001). We also found negative correlations between arm parameters and Overall BORG for parameters Overall_Arm_Length (*r* = −0.271, *p* < 0.001); Upper_Arm_Length (*r* = −0.151, *p* < 0.001); Lower_Arm_Length (*r* = −0.188, *p* < 0.001). For Finger_Length_Diff_3_5, a negative correlation was found with EMG Overall (*r* = −0.145, *p* < 0.05) but not with BORG Overall.

For parameters linked to passive spreading between fingers, negative correlations with Overall EMG were found for parameters Finger_Spreading_Passive_1_3 (*r* = −0.127, *p* < 0.05), Fingers_Spreading_Passive_2_3 (*r* = −0.142, *p* < 0.05), and Fingers_Spreading_Passive_3_4 (*r* = −0.126, *p* < 0.05).

## 5 Discussion

### 5.1 Instrument dimension effects

Results confirm a highly significant relationship between the instrument’s dimensions and subjectively perceived effort (BORG), but not for EMG at this data aggregation level, and for the total 16-s playing duration. One explanation for this discrepancy could be that EMG data is attributed to specific muscles along the upper left limb, whilst BORG Overall values summarize effort levels of the whole arm and hand. Another possible aspect worthy of future study might be that, due to previous playing experience, study participants have training-related, predictive motor commands. We hypothesize that these could be caused by feedforward control mechanisms of the central nervous system (Kozlowski et al., 2021; Morree de et al., 2012; Rewitz et al., 2024), allowing one to anticipate the level of effort while playing a given instrument. Effort anticipation might be linked to a reflex-like adaptation and recalibration of muscle activation levels with the aim of prolonging the time until muscular fatigue is reached, thereby optimizing performance efficacy and overall workload. An analysis of how and to what degree an instrument’s dimension or positioning elicits muscle activation and triggers an effort response within the context of such feedforward mechanisms may be considered an important future topic for research.

Future sub-analysis should allow us to understand if effects already observed for BORG will surface in EMG data when focusing on time sections within the tune under test, such as when using specific fingers, as well as when comparing results for the tests when the tune was played in 6th versus 2nd hand position (cf. section 3.6 above). Even though the difference between BORG and EMG values for the laboratory instruments V4 and V1 under test is smaller than what we can see when looking at results for instrument positioning effects (see below), it is noteworthy that study participants do feel clear differences between effort levels when playing the two instruments (V4 > V1).

From a practical point of view, acknowledging a player’s ability to distinguish between different degrees of effort may be beneficial in situations such as when choosing an individually appropriate instrument from an ergonomic perspective. Making such comparisons possible may be a valuable contribution to reducing the risk of developing Playing-Related Musculoskeletal Disorders (PRMDs) linked to the consequences of a less optimal fit between the player and the instrument.

### 5.2 Instrument positioning effects

Results confirm that instrument positioning effects of a viola are highly likely to affect both muscle activation (EMG) and subjectively perceived effort (BORG) in viola players. At the level of muscle activation (EMG), data collected for the viola shows a pattern much comparable to our previous research for violin (cf. Hildebrandt et al., 2021; Margulies et al., 2023): the gradation of muscle activation levels in the viola study shows highest levels for the A1 position, followed by the A2, the B1, and finally the B2 position for the laboratory instruments, and slight uptick in activation when the violists play on their own instrument (for details on positioning, please refer to Table 2 above). This pertains both to aggregated EMG data (“Overall EMG”), but also and in particular to the results at the single-muscle level for the *pectoralis major* as well as the *biceps brachii, caput breve* muscle. EMG as well as BORG effort levels of our datasets suggest that, despite players having chosen a positioning of their own instrument in proximity of B2 activation and effort levels, there appears to be potential for further reduction in muscular workload.

At the level of subjectively perceived effort (BORG), the gradation of values is also reconfirmed as in the violin study (cf. Hildebrandt et al., 2021; Margulies et al., 2023), but in the viola study, a new finding is that the instrument’s dimensions significantly affect this parameter in addition to positioning effects. This is an important indication that the aspect of the instrument’s build should be considered as an additional, relevant parameter, not only when playing but also when individually tailoring the choice of an instrument.

### 5.3 Positioning effects are more immediate than dimension effects

Results show that, both for muscle activation and subjectively perceived effort, the instrument positioning effects appear to outweigh the effects generated by the instrument’s dimensions. The V1-viola (small body and short string length) played in the A1 position yields significantly higher levels of muscle activation and perceived effort than V4-viola (large body and long string length) played in the B2 position: For EMG, this difference becomes visible already at highest levels of data aggregation, and – more pronouncedly – at single-muscle level for the *pectoralis major*, as well as the *biceps brachii caput breve,* muscle, as can be seen in results in section 4.3.1, where highly significant differences between the A1 and the B2 position were confirmed for these two muscles. This could be seen as an indication that in these position changes lies – if needed – the greatest potential for alleviation in terms of muscular workload and perceived effort as opposed to the more distally located muscles and reconfirms our findings for the violin (cf. Hildebrandt et al., 2021; Margulies et al., 2023). A new finding regarding the more distally located muscles is discussed below (cf. section “Biomechanics”).

Based on our data, these results bear quite some practical significance: When in need of reducing the levels of muscle activation and perceived effort (also as a temporary measure, e.g., when treating PRMDs in a violist), relief through position change can be given, as it were, instantly, even if a player currently uses a larger instrument. More complex solutions, such as modifying the instrument ergonomically by adapting the string length, or transitioning to a different, more suitable instrument, can then be sought in the next step.

### 5.4 Biomechanics

Among the results, a considerable percentage of the additional biomechanical parameters observed (cf. 3.1.4) show significant or highly significant correlations with muscle activation and subjectively perceived effort. The results for the correlation between the overall arm length and its parts (parameters Overall_Arm_Length, Upper_Arm_Length, Lower_Arm_Length) are one of our focal points of attention when considering the instrument’s build and dimensions (especially the instrument’s length) and their effects on the player. In these results, we see a first indication that an individual’s predisposition at the level of the arm may be a factor worth considering when looking at an optimal fit between the instrument and the player. Data suggests that the shorter the arm length and parts of it, the higher both muscle activation and subjectively perceived effort will be These results may become even more relevant in future sub-analysis, when examining data for instrument effects with a specific focus on the hand positions, in which the tune was played (i.e., 2nd vs. 6th position) and specific moments within the tune (i.e., if the use specific fingers such as the small finger impact data and how).

This also applies to the biomechanical parameters of active and passive supination. For our results at the highest aggregation level, it was a surprising finding that a negative correlation between active and passive supination and muscle activation was recorded for the more distally located *flexor carpi ulnaris* muscle, as opposed to the more proximally located *pectoralis major* muscle in the violin study (cf. Hildebrandt et al., 2021). One possible explanation for this shift could be that the violas under test were larger, both in their instrument body length and the vibrating string length, compared to the violin. We assume that the larger instrument dimensions may have influenced the degree of muscle activation in the more distally located muscles measured due to larger angles between the upper and lower arm and between the upper arm and the torso. This may be an explanation for an increase in leverage, respectively, the work of the arm against gravity, in combination with higher degrees of finger spreading due to the vibrating string length. This, in turn, may be considered as a factor leading to an increased workload at the level of the finger and wrist musculature. However, as with the arm length parameters above, future sub-analysis of data will show whether the hypothesized negative correlation between active and passive supination and muscle activation may still surface when examining data at lower aggregation levels (and hence in more detail as described above). This may become even more important, as currently at the highest data aggregation levels, we did, however, find a negative correlation between the parameter Supination_ Passive_250 and subjectively perceived effort (BORG Overall). This can be seen as an indication that study participants did feel that a lesser degree of passive supination ability at the finest torque level (a parameter identified as highly relevant for performance, cf. Wagner, 2005, 2012; Hildebrandt et al., 2021), increases the level of effort perceived while playing.

Reflecting our results against the background of the pre-existing body of literature poses a challenge known latest since the publication of a systematic review by (Chi et al. 2020), who confirm that the research methodologies aiming to give insight into the association between muscle activation and typical musculoskeletal disorders and musician’s anthropometrics and the instrument size or set-up are characterized by a high degree of heterogeneity. This applies, for example, to how electromyographic and anthropometric data were collected and interpreted, and how the aspect of ergonomics (such as the set-up with chinrest and shoulder pad) was included in the study design. Also, the broad spectrum of body regions in focus of a given study, as well as the outcome measures, pose challenges for comparability.

Notwithstanding this fact, a detailed analysis of the existing literature does permit us to establish some linkage between other research teams’ findings and this study. One such finding is that our results for the association between the left upper arm length and its parts and the levels of muscle activation when playing violas of differing dimensions and positioning resonate with results offered by Mann et al. (2021). Although our study did not include the bow hand and arm for methodological reasons, their results resonate well with ours in that they point to the fact that pragmatic alleviations achievable by, e.g., positional changes of the instrument may well be counted toward effective measures to influence (and if necessary reduce) the degree of muscle activation in the left upper limb when playing the instrument.

Possibly, the viola sizes (larger than a violin) as well as their positioning effects may be seen as mediator variables yielding the significant results. Future studies could serve as an opportunity to collect fresh anthropometric data for these same variables for the violin, which in our first study (cf. Margulies et al., 2023) had not been included.

Within the framework of our study, four muscles of the left upper limb (two more distally and two more proximally) were selected for surface EMG measurement, showing that the more proximal muscles (i.e., those more closely linked to compensatory movements of the left upper arm; cf. Margulies et al., 2023) are likely to see a higher degree of muscle activation than the distal muscles, depending on the instrument and its position chosen. In their study focusing on the left upper trapezius muscle, Berque and Gray (2002) suggest a redistribution mechanism of muscle activation to other synergistic muscles involved in the playing motion to alleviate that specific muscle’s workload. It could be assumed that the *biceps brachii and pectoralis major* muscle (focused on in our study as representants within the context of playing motions) elicit a response of the shoulder girdle musculature, such as the upper trapezius, infraspinatus and triceps muscle when adapting to the instrument’s size and positioning, thus contributing to the explanation of the development of musculoskeletal disorders PRMDs in the left shoulder girdle musculature (McCrary et al., 2016; Möller et al., 2018; Moore et al., 2008; Rensing et al., 2018). Therefore, our results add to the existing knowledge of the complex interaction of muscle “trains” from distal to proximal muscles and compensatory patterns in playing, considering instrument build and positioning as relevant factors. Future research will be necessary to focus on the aspect of compensatory playing movements. This will also permit us to examine more closely the role of active and passive supination ability and their influence on playing motion and muscle activation in viola playing.

Orchestra musicians playing instruments requiring an elevated arm position had a higher prevalence of neck-shoulder pain compared to their colleagues playing in a neutral arm position (Nyman et al., 2007). The results of this study on the viola and previous studies on the violin (Hildebrandt et al, 2021; Margulies et al., 2023) could help musicians and health practitioners to reduce or rebalance muscular tension in the shoulder girdle and arms by means of individually adapting the instrument’s positioning. Also, the choice of an instrument uniting optimal acoustic characteristics with individually tailored, ergonomic features would benefit the player despite the required elevated arm position.

Such optimizations can be seen as an important element in view of preventing PRMDs, such as the shoulder impingement syndrome, to which all muscles bringing the humerus closer to the acromion can contribute (e.g., the pectoral, bicep, and triceps as well as the deltoid muscles). In their paper of 2008, Moore et al. identified players of high-stringed instruments as being at increased risk for shoulder impingement (Moore et al., 2008). This fits well with our findings both for the violin and for the viola, as the *biceps brachii* and *pectoralis major muscles* increase tension most obviously depending on hand and arm positions in playing contexts and therefore could play an important role in the development of impingement syndromes. Other authors have pointed out the relevance of the instrument’s size relative to its effect on the shoulder girdle musculature and the rotator cuff (Blum, 1993, 1995a, 1995b; Blum and Ahlers 1994; Moog, 1995). Hence, our study can be seen as a contribution to the explanation for the emergence of such syndromes in players of high-stringed instruments.

## 6 Conclusion

As first observed in our research dedicated to the violin, this project has confirmed that positional changes of the instrument significantly affect muscle activation (EMG) and subjectively perceived effort (BORG), and that, at this level of data aggregation, positioning effects are clearer than effects coming from an instrument’s dimensions. We have also shown that the association between EMG/BORG and instrument positioning is stronger for the more proximally located muscles examined, and that there is a relevant association between arm length parameters with EMG and BORG. A key takeaway from a practical standpoint is that muscle activation levels and effort perception can be significantly reduced by means of positional changes of the instrument, even if it were largely dimensioned. Data suggests that this can lead to a greater degree of ease compared to when using a smaller dimensioned instrument in a physiologically less favorable position. In sum, results permit expanding existing knowledge and experience for the violin to the viola. A special focus thereby could lie on deducing ergonomic solutions considering the impact of the instrument’s orientation (i.e., the individualized definition and choice of the instrument’s longitudinal as well as transversal axis relative to the player; cf. Hildebrandt et al., 2021; Margulies et al., 2023), but also the consideration of potential effects of the way the instrument is built (overall size and vibrating string length). This could likely benefit players (professionals and non-professionals alike) in preventing task-specific health problems and in optimizing and increasing their performance. Findings contribute to the growing body of science-based approaches to deal efficiently with PRMDs and also to help players of high-stringed instruments to thrive through music educators’ processing and integrating our results and insights into their teaching, as well as the broad spectrum of healthcare professionals.

### 6.1 Limitations

This study was carried out under laboratory conditions and without the study participants using their bow in view of reducing the risk of confounding variables. Further studies will be needed to compare current results with datasets generated when players perform a tune with the bow. Further research will also be needed to understand how longer durations of playing affect muscle activation and subjectively perceived effort. Longitudinal studies going beyond initial laboratory findings would also be required to gauge how the effects observed within the framework of this study can be transferred into real-world settings.

## Data Availability

The datasets presented in this study can be found in online repositories. The names of the repository/repositories and accession number(s) can be found below: https://zenodo.org/records/15243357.
