# Supplementary material for: Viola ergonomics for thriving and health promotion: the influence of an instrument’s dimensions, positioning, and biomechanics on muscular effort
Source: Front Psychol. 2026 Apr 10;17:1767988. doi: 10.3389/fpsyg.2026.1767988 (PMC13107652; doi:10.3389/fpsyg.2026.1767988)
Supplement: Supplementary file 1 [file Data_Sheet_1.PDF]

Supplementary Material

Table S1

Parameter	Norm ranges viola [mm]*	Norm ranges violin [mm]*
**Overall length**	640 – 730 (Δ 90)	586 – 609 (Δ 23)
**Body length**	380 – 435 (Δ 55)	352 – 369 (Δ 17)
**Max. width lower bout**	230 – 270 (Δ 40)	198 – 211 (Δ 13)
**Max. width upper bout**	180 – 220 (Δ 40)	159 – 170 (Δ 11)
**Min. width at waist**	116 – 135 (Δ 19)	102 – 113 (Δ 11)
**Height of body**	63 – 89 (Δ 25)	56 – 69 (Δ 13)
**string length**	350 – 400 (Δ 50)	324 – 331 (Δ 7)

Table S1: Comparison between norm ranges of the viola vs. the violin (*Data curtesy to Wilhelm Geigenbau)

Small-dimensioned instruments for children, over-dimensioned instruments (e.g. the "Ritter" viola) as well as the viola's historic precursors (e.g. the viola da braccio, viola da spalla, viola bastarda and viola d'amore) are not included in this table, as these instruments would require isolated research questions specifically for this instrument due to their distinct differences from the norm.

Table S2: Multiple comparison of means (Scheffé) by instrument (V1, V4, Free) for EMG Overall and by single channel

EMG Channel	Instrument	Instrument comparison	Difference of mean	SE	Significance	95% Confidence interval	
						Lower margin	Upper margin
**EMG Overall (4 channels)**	**V1**	**V4**	-0.201	0.160	0.457	-0.595	0.193
		**Free**	0.142	0.254	0.856	-0.481	0.764
	**V4**	**V1**	0.201	0.160	0.457	-0.193	0.595
		**Free**	0.343	0.254	0.402	-0.280	0.966
	**Free**	**V1**	-0.142	0.254	0.856	-0.764	0.481
		**V4**	-0.343	0.254	0.402	-0.966	0.280
**EMG Channel A (M. pectoralis major)**	**V1**	**V4**	-0.154	0.191	0.723	-0.623	0.315
		**Free**	0.738	0.302	0.051	-0.003	1.479
	**V4**	**V1**	0.154	0.191	0.723	-0.315	0.623
		**Free**	0.892*	0.302	0.013	0.150	1.633
	**Free**	**V1**	-0.738	0.302	0.051	-1.479	0.003
		**V4**	-0.892	0.302	0.013	-1.633	-0.150
**EMG Channel B (M. biceps brachii, caput breve)**	**V1**	**V4**	-0.315	0.267	0.500	-0.971	0.341
		**Free**	-0.105	0.423	0.970	-1.142	0.932
	**V4**	**V1**	0.315	0.267	0.500	-0.341	0.971
		**Free**	0.210	0.423	0.884	-0.827	1.247
	**Free**	**V1**	0.105	0.423	0.970	-0.932	1.142
		**V4**	-0.210	0.423	0.884	-1.247	0.827
**EMG Channel C (M. extensor carpi ulnaris)**	**V1**	**V4**	-0.246	0.244	0.601	-0.845	0.352
		**Free**	-0.358	0.386	0.650	-1.304	0.588
	**V4**	**V1**	0.246	0.244	0.601	-0.352	0.845
		**Free**	-0.112	0.386	0.959	-1.058	0.834
	**Free**	**V1**	0.358	0.386	0.650	-0.588	1.304
		**V4**	0.112	0.386	0.959	-0.834	1.058
**EMG Channel D (M. extensor digitorum communis)**	**V1**	**V4**	-0.089	0.150	0.838	-0.458	0.279
		**Free**	0.292	0.237	0.469	-0.290	0.875
	**V4**	**V1**	0.089	0.150	0.838	-0.279	0.458
		**Free**	0.382	0.237	0.275	-0.201	0.964
	**Free**	**V1**	-0.292	0.237	0.469	-0.875	0.290
		**V4**	-0.382	0.237	0.275	-0.964	0.201

Table S2: Multiple comparison of means (Scheffé) by instrument (V1, V4, Free) for EMG Overall and by single channel

Tables S3a, S3b, and S3c (EMG by instrument position)

Table S3a: Descriptive statistics

Instrument Position	Mean, number of observations, standard deviation	EMG Overall (4 channels)	EMG Channel A (M. pectoralis major)	EMG Channel B (M. biceps brachii, caput breve)	EMG Channel C (M. extensor carpi ulnaris)	EMG Channel D (M. extensor digitorum communis)
A1	Mean	4.133	4.092	5.365	3.486	3.589
	N	124	124	124	124	124
	SD	2.027	2.564	3.669	2.814	1.824
A2	Mean	3.552	3.395	4.341	3.197	3.274
	N	124	124	124	124	124
	SD	1.688	2.039	2.621	2.698	1.638
B1	Mean	3.355	2.734	4.139	3.179	3.366
	N	124	124	124	124	124
	SD	1.708	1.952	2.700	2.770	1.713
B2	Mean	2.925	2.104	3.454	3.065	3.076
	N	124	124	124	124	124
	SD	1.523	1.505	2.309	2.705	1.559
Free	Mean	3.249	2.267	4.273	3.467	2.989
	N	62	62	62	62	62
	SD	1.732	1.693	3.242	2.475	1.495
Insgesamt	Mean	3.464	2.991	4.319	3.258	3.289
	N	558	558	558	558	558
	SD	1.788	2.140	2.974	2.714	1.673

Table S3b: ANOVA

			Sum of Squares	df	Mean of Squares	F	Sig.
EMG Overall (4 channels) * Position	Between groups	(combined)	96.847	4	24.212	7.953	0.000
	Witin groups		1683.492	553	3.044		
	Overall		1780.339	557			
EMG Channel A (M. pectoralis major) * Position	Between groups	(combined)	308.881	4	77.220	19.043	0.000
	Witin groups			553	4.055		
	Overall			557			
EMG Channel B (M. biceps brachii, caput breve) * Position	Between groups	(combined)	232.667	4	58.167	6.852	0.000
	Witin groups			553	8.489		
	Overall			557			
EMG Channel C (M. extensor carpi ulnaris) * Position	Between groups	(combined)	14.993	4	3.748	0.507	0.731
	Witin groups			553	7.391		
	Overall			557			
EMG Channel D (M. extensor digitorum communis) * Position	Between groups	(combined)	23.083	4	5.771	2.078	0.082
	Witin groups			553	2.777		
	Overall			557			

Table S3c: Multiple comparison of means (Scheffé)

						95% Confidence interval	
EMG Channel	Instrume nt Position	Instrument Position Comparison	Difference of Mean	SE	Sig.	Lower Margin	Upper Margin
EMG Overall Mean (4 channels)	A1	A2	0.581	0.222	0.144	-0.104	1.266
		B1	,778*	0.222	0.016	0.093	1.463
		B2	1,208*	0.222	0.000	0.523	1.893
		Free	,884*	0.271	0.032	0.045	1.723
	A2	A1	-0.581	0.222	0.144	-1.266	0.104
		B1	0.197	0.222	0.939	-0.488	0.882
		B2	0.627	0.222	0.093	-0.058	1.312
		Free	0.303	0.271	0.870	-0.536	1.142
	B1	A1	,778*	0.222	0.016	-1.463	-0.093
		A2	-0.197	0.222	0.939	-0.882	0.488
		B2	0.430	0.222	0.440	-0.255	1.115
		Free	0.106	0.271	0.997	-0.733	0.945
	B2	A1	1,208*	0.222	0.000	-1.893	-0.523
		A2	-0.627	0.222	0.093	-1.312	0.058
		B1	-0.430	0.222	0.440	-1.115	0.255

		**Free**	-0.324	0.271	0.840	-1.163	0.515
	**Free**	**A1**	-,884*	0.271	0.032	-1.723	-0.045
		**A2**	-0.303	0.271	0.870	-1.142	0.536
		**B1**	-0.106	0.271	0.997	-0.945	0.733
		**B2**	0.324	0.271	0.840	-0.515	1.163
**EMG Channel A (M. pectoralis major):** Mean	**A1**	**A2**	0.697	0.256	0.116	-0.093	1.488
		**B1**	1,359*	0.256	0.000	0.568	2.149
		**B2**	1,988*	0.256	0.000	1.198	2.778
		**Free**	1,826*	0.313	0.000	0.858	2.794
	**A2**	**A1**	-0.697	0.256	0.116	-1.488	0.093
		**B1**	0.661	0.256	0.155	-0.129	1.452
		**B2**	1,291*	0.256	0.000	0.500	2.081
		**Free**	1,1284*	0.313	0.012	0.160	2.097
	**B1**	**A1**	-1,359*	0.256	0.000	-2.149	-0.568
		**A2**	-0.661	0.256	0.155	-1.452	0.129
		**B2**	0.629	0.256	0.197	-0.161	1.420
		**Free**	0.467	0.313	0.695	-0.501	1.435
	**B2**	**A1**	-1,988*	0.256	0.000	-2.778	-1.198
		**A2**	-1,291*	0.256	0.000	-2.081	-0.500
		**B1**	-0.629	0.256	0.197	-1.420	0.161
		**Free**	-0.162	0.313	0.992	-1.130	0.806
	**Free**	**A1**	-1,826*	0.313	0.000	-2.794	-0.858
		**A2**	-1,128*	0.313	0.012	-2.097	-0.160
		**B1**	-0.467	0.313	0.695	-1.435	0.501
		**B2**	0.162	0.313	0.992	-0.806	1.130
**EMG Channel B (M. biceps brachii, caput breve):** Mean	**A1**	**A2**	1.024	0.370	0.106	-0.120	2.168
		**B1**	1,226*	0.370	0.028	0.082	2.370
		**B2**	1,911*	0.370	0.000	0.767	3.055
		**Free**	1.093	0.453	0.215	-0.308	2.493
	**A2**	**A1**	-1.024	0.370	0.106	-2.168	0.120
		**B1**	0.202	0.370	0.990	-0.942	1.346
		**B2**	0.887	0.370	0.221	-0.257	2.031
		**Free**	0.068	0.453	1.000	-1.332	1.469
	**B1**	**A1**	-1,226*	0.370	0.028	-2.370	-0.082
		**A2**	-0.202	0.370	0.990	-1.346	0.942
		**B2**	0.685	0.370	0.490	-0.459	1.829
		**Free**	-0.133	0.453	0.999	-1.534	1.267
	**B2**	**A1**	-1,911*	0.370	0.000	-3.055	-0.767
		**A2**	-0.887	0.370	0.221	-2.031	0.257
		**B1**	-0.685	0.370	0.490	-1.829	0.459
		**Free**	-0.818	0.453	0.516	-2.219	0.582
	**Free**	**A1**	-1.093	0.453	0.215	-2.493	0.308
		**A2**	-0.068	0.453	1.000	-1.469	1.332
		**B1**	0.133	0.453	0.999	-1.267	1.534
		**B2**	0.818	0.453	0.516	-0.582	2.219
**EMG Channel C (M. extensor carpi ulnaris):** Mean	**A1**	**A2**	0.288	0.345	0.951	-0.779	1.356
		**B1**	0.306	0.345	0.940	-0.761	1.373
		**B2**	0.421	0.345	0.829	-0.646	1.488
		**Free**	0.019	0.423	1.000	-1.288	1.326
	**A2**	**A1**	-0.288	0.345	0.951	-1.356	0.779
		**B1**	0.018	0.345	1.000	-1.049	1.085
		**B2**	0.133	0.345	0.997	-0.934	1.200
		**Free**	-0.270	0.423	0.982	-1.577	1.037
	**B1**	**A1**	-0.306	0.345	0.940	-1.373	0.761
		**A2**	-0.018	0.345	1.000	-1.085	1.049
		**B2**	0.115	0.345	0.999	-0.952	1.182
		**Free**	-0.287	0.423	0.977	-1.594	1.020
	**B2**	**A1**	-0.421	0.345	0.829	-1.488	0.646
		**A2**	-0.133	0.345	0.997	-1.200	0.934
		**B1**	-0.115	0.345	0.999	-1.182	0.952
		**Free**	-0.402	0.423	0.924	-1.709	0.905
	**Free**	**A1**	-0.019	0.423	1.000	-1.326	1.288
		**A2**	0.270	0.423	0.982	-1.037	1.577
		**B1**	0.287	0.423	0.977	-1.020	1.594
		**B2**	0.402	0.423	0.924	-0.905	1.709

**EMG Channel D (M. extensor digitorum communis):** Mean	**A1**	**A2**	0.315	0.212	0.697	-0.339	0.969
		**B1**	0.222	0.212	0.894	-0.432	0.876
		**B2**	0.512	0.212	0.211	-0.142	1.166
		**Free**	0.599	0.259	0.255	-0.202	1.400
	**A2**	**A1**	-0.315	0.212	0.697	-0.969	0.339
		**B1**	-0.092	0.212	0.996	-0.746	0.562
		**B2**	0.198	0.212	0.928	-0.456	0.852
		**Free**	0.285	0.259	0.877	-0.516	1.086
	**B1**	**A1**	-0.222	0.212	0.894	-0.876	0.432
		**A2**	0.092	0.212	0.996	-0.562	0.746
		**B2**	0.290	0.212	0.758	-0.364	0.944
		**Free**	0.377	0.259	0.715	-0.424	1.178
	**B2**	**A1**	-0.512	0.212	0.211	-1.166	0.142
		**A2**	-0.198	0.212	0.928	-0.852	0.456
		**B1**	-0.290	0.212	0.758	-0.944	0.364
		**Free**	0.087	0.259	0.998	-0.714	0.888
	**Free**	**A1**	-0.599	0.259	0.255	-1.400	0.202
		**A2**	-0.285	0.259	0.877	-1.086	0.516
		**B1**	-0.377	0.259	0.715	-1.178	0.424
		**B2**	-0.087	0.259	0.998	-0.888	0.714

Tables S4a, S4b, and S4c (BORG values by instrument position)

Table S4a: Descriptive statistics

Instrument Position	Mean, Number of observations, standard deviation	BORG Overall
A1	Mean	12.27
	N	124
	SD	3.162
A2	Mean	10.38
	N	124
	SD	2.686
B1	Mean	10.79
	N	124
	SD	2.907
B2	Mean	8.84
	N	124
	SD	2.245
Free	Mean	9.29
	N	62
	SD	2.418

Table S4b: ANOVA

			Sum of Squares	df	Mean of Squares	F	Sig.
**BORG Overall * Position**	**Between groups**	**(combined)**	829.556	4	207.389	27.747	0.000
	**Witin groups**			553	7.474		
	**Overall**			557			

Table S4c: Multiple comparison of means (Scheffé)

					95% Confidence interval	
Instrument Position	Instrument Position Comparison	Difference of Mean	SE	Sig.	Lower Margin	Upper Margin
A1	A2	1,887*	0.347	0.000	0.81	2.96
	B1	1,476*	0.347	0.001	0.40	2.55
	B2	3,427*	0.347	0.000	2.35	4.50
	Free	2,980*	0.425	0.000	1.67	4.29

Tables S5a, S5b, and S5c (EMG by instrument position for subanalysis Instrument V1/Instrument position A1 vs. instrument V4/instrument position B2)

Table S5a: Descriptive statistics for EMG

Combination Instrument, Instrument Position	Mean, number of observations, standard deviation	EMG Overall (4 channels)	EMG Channel A (M. pectoralis major)	EMG Channel B (M. biceps brachii, caput breve)	EMG Channel C (M. extensor carpi ulnaris)	EMG Channel D (M. extensor digitorum communis)
**V1, A1**	**Mean**	3.983	4.039	5.072	3.305	3.516
	**N**	62	62	62	62	62
	**SD**	1.937	2.547	3.350	2.781	1.782
**V1, A2**	**Mean**	3.445	3.321	4.150	3.088	3.221
	**N**	62	62	62	62	62
	**SD**	1.618	2.037	2.349	2.838	1.614
**V1, B1**	**Mean**	3.262	2.644	4.028	3.056	3.321
	**N**	62	62	62	62	62
	**SD**	1.715	1.908	2.549	2.859	1.800
**V1, B2**	**Mean**	2.872	2.014	3.420	2.986	3.069
	**N**	62	62	62	62	62
	**SD**	1.593	1.458	2.363	2.816	1.611
**V4, A1**	**Mean**	4.283	4.146	5.658	3.667	3.662
	**N**	62	62	62	62	62
	**SD**	2.119	2.602	3.968	2.858	1.876
**V4, A2**	**Mean**	3.659	3.469	4.532	3.306	3.327
	**N**	62	62	62	62	62
	**SD**	1.762	2.054	2.875	2.569	1.673
**V4, B1**	**Mean**	3.447	2.824	4.250	3.303	3.412
	**N**	62	62	62	62	62
	**SD**	1.711	2.007	2.860	2.696	1.635
**V4, B2**	**Mean**	2.978	2.195	3.489	3.144	3.084
	**N**	62	62	62	62	62
	**SD**	1.462	1.557	2.273	2.610	1.519
**Free**	**Mean**	3.249	2.267	4.273	3.467	2.989
	**N**	62	62	62	62	62
	**SD**	1.732	1.693	3.242	2.475	1.495
**Overall**	**Mean**	3.464	2.991	4.319	3.258	3.289
	**N**	558	558	558	558	558
	**SD**	1.788	2.140	2.974	2.714	1.673

Table S5b: ANOVA

			Sum of Squares	df	Mean of Squares	F	Sig.
**EMG Overall (4 channels)** * combination: Instrument (V1,V4,Free, all=0), and Position(a1,a2,b1,b2,free,all=0)	**Between groups**	**(combined)**	102.458	8	12.807	4.191	0.000
	**Witin groups**			549	3.056		
	**Overall**			557			
**EMG Channel A (M. pectoralis major)** * combination: Instrument (V1,V4,Free, all=0), and Position(a1,a2,b1,b2,free,all=0)	**Between groups**	**(combined)**	311.931	8	38.991	9.559	0.000
	**Witin groups**			549	4.079		
	**Overall**			557			
**EMG Channel B (M. biceps brachii, caput breve)** * combination: Instrument (V1,V4,Free, all=0), and Position(a1,a2,b1,b2,free,all=0)	**Between groups**	**(combined)**	249.516	8	31.189	3.661	0.000
	**Witin groups**			549	8.520		
	**Overall**			557			
**EMG Channel C (M. extensor carpi ulnaris)** * combination: Instrument (V1,V4,Free, all=0), and Position(a1,a2,b1,b2,free,all=0)	**Between groups**	**(combined)**	23.190	8	2.899	0.390	0.926
	**Witin groups**			549	7.430		
	**Overall**			557			
**EMG Channel D (M. extensor digitorum communis)** * combination: Instrument (V1,V4,Free, all=0), and Position(a1,a2,b1,b2,free,all=0)	**Between groups**	**(combined)**	24.353	8	3.044	1.089	0.369
	**Witin groups**			549	2.794		
	**Overall**			557			

Table S5c: Multiple comparison of means (Scheffé), for all muscles

					95% Confidence interval	
**Combination Instrument, instrument position**	**Instrument Position Comparison**	**Difference of Mean**	**SE**	**Sig.**	**Lower Margin**	**Upper Margin**
V1, A1	V1, A2	0.538	0.314	0.938	-0.704	1.780
	V1, B1	0.721	0.314	0.728	-0.521	1.962
	V1, B2	1.111	0.314	0.132	-0.131	2.353
	V4, A1	-0.300	0.314	0.999	-1.542	0.942
	V4, A2	0.324	0.314	0.998	-0.918	1.566
	V4, B1	0.536	0.314	0.939	-0.706	1.778
	V4, B2	1.005	0.314	0.251	-0.237	2.247
	Free	0.734	0.314	0.707	-0.508	1.976

Table S5d: Multiple comparison of means (Scheffé), for pectoralis major muscle

					95% Confidence interval	
**Combination Instrument, instrument position**	**Instrument Position Comparison**	**Difference of Mean**	**SE**	**Sig.**	**Lower Margin**	**Upper Margin**
V1, A1	V1, A2	0.719	0.363	0.863	-0.716	2.153
	V1, B1	1.396	0.363	0.066	-0.039	2.830
	V1, B2	2,025*	0.363	0.000	0.591	3.460
	V4, A1	-0.106	0.363	1.000	-1.541	1.328
	V4, A2	0.570	0.363	0.963	-0.864	2.005
	V4, B1	1.216	0.363	0.192	-0.219	2.650
	V4, B2	1,844*	0.363	0.001	0.410	3.279
	Free	1,773*	0.363	0.003	0.338	3.207

Table S5e: Multiple comparison of means (Scheffé), for biceps brachii muscle

					95% Confidence interval	
**Combination Instrument, instrument position**	**Instrument Position Comparison**	**Difference of Mean**	**SE**	**Sig.**	**Lower Margin**	**Upper Margin**
V1, A1	V1, A2	0.922	0.524	0.928	-1.152	2.995
	V1, B1	1.044	0.524	0.860	-1.030	3.117
	V1, B2	1.652	0.524	0.272	-0.421	3.726
	V4, A1	-0.586	0.524	0.996	-2.660	1.487
	V4, A2	0.540	0.524	0.998	-1.533	2.614
	V4, B1	0.822	0.524	0.963	-1.251	2.895
	V4, B2	1.583	0.524	0.334	-0.490	3.657
	Free	0.799	0.524	0.969	-1.274	2.873

Tables S6a-c (BORG by instrument position for sub-analysis Instrument V1/Instrument position A1 vs. instrument V4/instrument position B2)

Table S6a: Descriptive statistics

Combination: Instrument (V1,V4,Free, all=0), and Position(a1,a2,b1,b2,free,all=0)	Mean, Number of observations, standard deviation	BORG Overall
V1, A1	Mean	11.85
	N	62
	SD	3.062
V1, A2	Mean	10.12
	N	62
	SD	2.676
V1, B1	Mean	10.27
	N	62
	SD	2.609
V1, B2	Mean	8.56
	N	62
	SD	2.146
V4, A1	Mean	12.69
	N	62
	SD	3.228
V4, A2	Mean	10.65
	N	62
	SD	2.692
V4, B1	Mean	11.32
	N	62
	SD	3.109
V4, B2	Mean	9.13
	N	62
	SD	2.320
Free	Mean	9.29
	N	62
	SD	2.418
Overall	Mean	10.43
	N	558
	SD	2.985

Table S6b: Anova

			Sum of Squares	df	Mean of Squares	F	Sig.
BORG Overall * combination: Instrument (V1,V4,Free, all=0), and Position(a1,a2,b1,b2,free,all=0)	Between groups	(combined)	905.065	8	113.133	15.307	0.000
	Witin groups			549	7.391		
	Overall			557			

Table S6b: Multiple comparison of means (Scheffé)

Combination Instrument, instrument position	Instrument, Instrument Position Comparison	Difference of Mean	SE	Sig.	95% Confidence interval Lower Margin	Upper Margin
**V1, A1**	**V1, A2**	1.726	0.488	0.133	-0.21	3.66
	**V1, B1**	1.581	0.488	0.236	-0.35	3.51
	**V1, B2**	3,290*	0.488	0.000	1.36	5.22
	**V4, A1**	-0.847	0.488	0.933	-2.78	1.08
	**V4, A2**	1.202	0.488	0.641	-0.73	3.13
	**V4, B1**	0.524	0.488	0.997	-1.41	2.46
	**V4, B2**	2,718*	0.488	0.000	0.79	4.65
	**Free**	2,556*	0.488	0.001	0.63	4.49
